# Engineered Fusion Enzyme‐Mediated Non‐Consecutive Cyclization‐Glycosylation Enables Heterologous Synthesis of Antifungal Enfumafungin

**DOI:** 10.1002/advs.202507531

**Published:** 2025-09-02

**Authors:** Yaohui Gao, Jianming Lv, Yue Zhong, Zhiqin Cao, Rui Luo, Yue Qi, Gaoqian Wang, Shaoyang Li, Guodong Chen, Dan Hu, Hao Gao, Xinsheng Yao

**Affiliations:** ^1^ School of Traditional Chinese Materia Medica Shenyang Pharmaceutical University Shenyang 110016 China; ^2^ Institute of Traditional Chinese Medicine and Natural Products, College of Pharmacy/State Key Laboratory of Bioactive Molecules and Druggability Assessment/International Cooperative Laboratory of Traditional Chinese Medicine Modernization and Innovative Drug Development of Ministry of Education (MOE) of China/Guangdong Province Key Laboratory of Pharmacodynamic Constituents of Traditional Chinese Medicine and New Drugs Research Jinan University Guangzhou 510632 China; ^3^ Guangdong Provincial Key Laboratory of Natural Drugs Research and Development, School of Pharmacy Guangdong Medical University Dongguan 523808 China

**Keywords:** antifungal, fungi, fusion enzyme, heterologous expression, triterpenoid biosynthesis

## Abstract

Enfumafungin‐type antibiotics, represented by enfumafungin and fuscoatroside, constitute a distinct class of fungi‐derived fernane‐type triterpenoids renowned for their potent antifungal activity. Notably, ibrexafungerp, a semi‐chemically synthesized analogue of enfumafungin, has recently received approval as a novel antifungal drug. Thus, reconstituting the heterologous biosynthesis of enfumafungin holds great significance, as it offers a promising route for high‐level production. Herein, the *Aspergillus oryzae* S184 chassis is first optimized. By deleting *ku80* gene and refining counter‐selection procedure, site‐specific gene integration and substantially shortened the time required for selection marker recycling are significantly enhanced. Subsequently, an artificial biosynthetic pathway potentially involved in enfumafungin biosynthesis is successfully reconstructed. Crucially, the native terpene cyclase (TC)‐glycosyltransferase (GT) fusion enzyme, EfuA, involved in enfumafungin biosynthesis, lost its functionality in *A. oryzae*. Conversely, a designed fusion enzyme EfuA_(TC)_FsoA_(GT)_, which combines the TC domain of EfuA with the GT domain of FsoA (involved in fuscoatroside biosynthesis), along with FsoD/E/F, efficiently produced the putative enfumafungin intermediate. The functional analysis further revealed that while the fusion of the TC and GT domains is critical for maintaining dual enzymatic activity, these fusion enzymes catalyze unconventional, non‐consecutive terpene cyclization and glycosylation steps during the biosynthesis of enfumafungin‐type antibiotics, differing from other canonical fusion enzymes.

## Graphical abstract

The artificial biosynthetic pathway potentially involved in biosynthesis of enfumafungin is first reconstructed in an optimized *Aspergillus oryzae* chassis with the increased site‐specific integration efficiency and shortened time for marker recycling, which requires an artificial fusion enzyme EfuA_(TC)_FsoA_(GT)_. Crucially, the fusion enzymes, involved in biosynthesis of enfumafungin‐type antibiotics, catalyze non‐consecutive cyclization and glycosylation, different from canonical fusion enzymes.

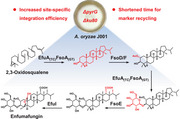

## 1 Introduction

Enfumafungin‐type antibiotics are a unique group of fungi‐derived fernane‐type triterpenoids. They are characterized by a cleaved E‐ring, a 3‐*O*‐*β*‐d‐glucopyranosyl group and a 2*α*‐hydroxyl group.^[^ ^1^ ^]^ Prominent members of this group include enfumafungin,^[^ ^2^ ^]^ fuscoatroside,^[^ ^3^ ^]^ kolokoside A,^[^ ^4^ ^]^ and WF11605 (Figure S1, Supporting Information).^[^ ^5^ ^]^ These antibiotics potently inhibit the *β*‐1,3‐glucan synthase, an enzyme crucial for fungal cell wall formation, thereby exhibiting potent antifungal activity.^[^ ^6^ ^]^ In particular, ibrexafungerp, a semisynthetic derivative of enfumafungin, was approved in 2021 by the U.S. Food and Drug Administration for treating invasive vulvovaginal candidiasis.^[^ ^7^ ^]^ Given their significance, elucidating the biosynthetic pathways of enfumafungin‐type antibiotics and achieving heterologous biosynthesis of enfumafungin in a chassis organism has attracted substantial attention. This approach holds great promise for large scale production of enfumafungin through subsequent metabolic engineering efforts.

In 2018, Kuhnert et al. first identified a putative biosynthetic gene cluster (*efu*) of enfumafungin, which contains an unusual terpene cyclase (TC)‐glycosyltransferase (GT) fusion gene *efuA*.^[^ ^8^ ^]^ Although they confirmed the involvement of this fusion gene in the biosynthesis of enfumafungin, its detailed function remains unclear. Subsequently, we demonstrated that the TC domain of EfuA is responsible for fernenol biosynthesis.^[^ ^9^ ^]^ More recently, we identified a candidate biosynthetic gene cluster (*fso*) for fuscoatroside from *Humicola fuscoatra* NRRL 22980. This cluster also includes a TC‐GT fusion enzyme gene *fsoA*, along with three cytochrome P450 enzyme genes *fsoB/D/E*, one acetyltransferase gene *fsoF*, and a glucose oxidase gene *fsoC*.^[^ ^1^ ^]^ Subsequently, through heterologous expression in *Aspergillus oryzae* NSAR1, we elucidated the biosynthetic pathway of fuscoatroside, which involves complex biosynthetic networks.^[^ ^1^ ^]^ Specifically, the TC domain of the bifunctional fusion enzyme FsoA catalyzes the cyclization of 2,3‐oxidosqualene to isomotiol, and the GT domain transfers the glucose moiety to 3‐OH; FsoD and FsoF are responsible for hydroxylation at C2 and acetylation of 2‐OH, respectively; FsoE triggers the cleavage of the E ring. Slightly different from fuscoatroside, enfumafungin is derived from fernenol, the double bond isomer of isomotiol, and bears an additional hemiacetal moiety formed between C24 and C25. Although the biosynthesis of enfumafungin remains poorly understood, insights into the fuscoatroside pathway provide a foundation for reconstructing enfumafungin biosynthesis in a heterologous host.

For decades, *Saccharomyces cerevisiae* has been used as the dominant host for heterologous expression of fungi‐derived biosynthetic genes, owing to its well‐established genetic manipulation system.^[^ ^10^ ^]^ In recent decades, the *Aspergillus* species emerge as an increasingly prevalent alternative,^[^ ^11^, ^12^ ^]^ which are not only able to precisely splice the introns of exogenous fungal genes, but also do not require additional cytochrome P450 reductases when cytochrome P450 monooxygenases are introduced. Previously, we have used quadruple auxotrophic *A. oryzae* NSAR1 and achieved the heterologous biosynthesis of several fungal triterpenoids,^[^ ^1^, ^9^, ^13^, ^14^, ^15^, ^16^ ^]^ implying the potential of *A. oryzae* NSAR1 for reconstituting enfumafungin biosynthesis. However, there are two drawbacks that prevent *A. oryzae* NSAR1 from serving as an ideal chassis. One issue is that the selection markers cannot be used iteratively, and the other is that the exogenous genes are randomly integrated into the genome without controlling of their copy number. Recently, Yuan et al. developed *A. oryzae* S184 by disrupting the orotidine‐5′‐monophosphate decarboxylase gene *(pyrG*) of *A. oryzae* NSAR1, which enables iterative introduction of multiple genes through the CRISPR‐Cas9‐based recyclable selection marker system.^[^ ^17^ ^]^ However, challenges such as the generation of many false‐positive clones and the long time required for marker recycling have limited its widespread adoption.

Here, we engineered *A. oryzae* S184 with an enhanced gene integration efficiency and an optimized counter‐selection system to reconstitute an artificial biosynthetic pathway potentially involved in the formation of enfumafungin. Notably, while the native TC‐GT fusion enzyme EfuA exhibited limited functionality in the heterologous host, our designed artificial fusion enzyme EfuA_(TC)_FsoA_(GT)_ demonstrated enhanced catalytic efficiency for producing the putative enfumafungin intermediate. Crucially, our functional analysis revealed that although the fusion of TC and GT domains is critical for maintaining the dual enzymatic activity, these fusion enzymes catalyze unusual non‐consecutive terpene cyclization and glycosylation steps in the biosynthesis of enfumafungin‐type antibiotics.

## 2 Results and Discussion

### 2.1 Optimization of the *A. oryzae* S184 Heterologous Expression System

**Figure 1:**
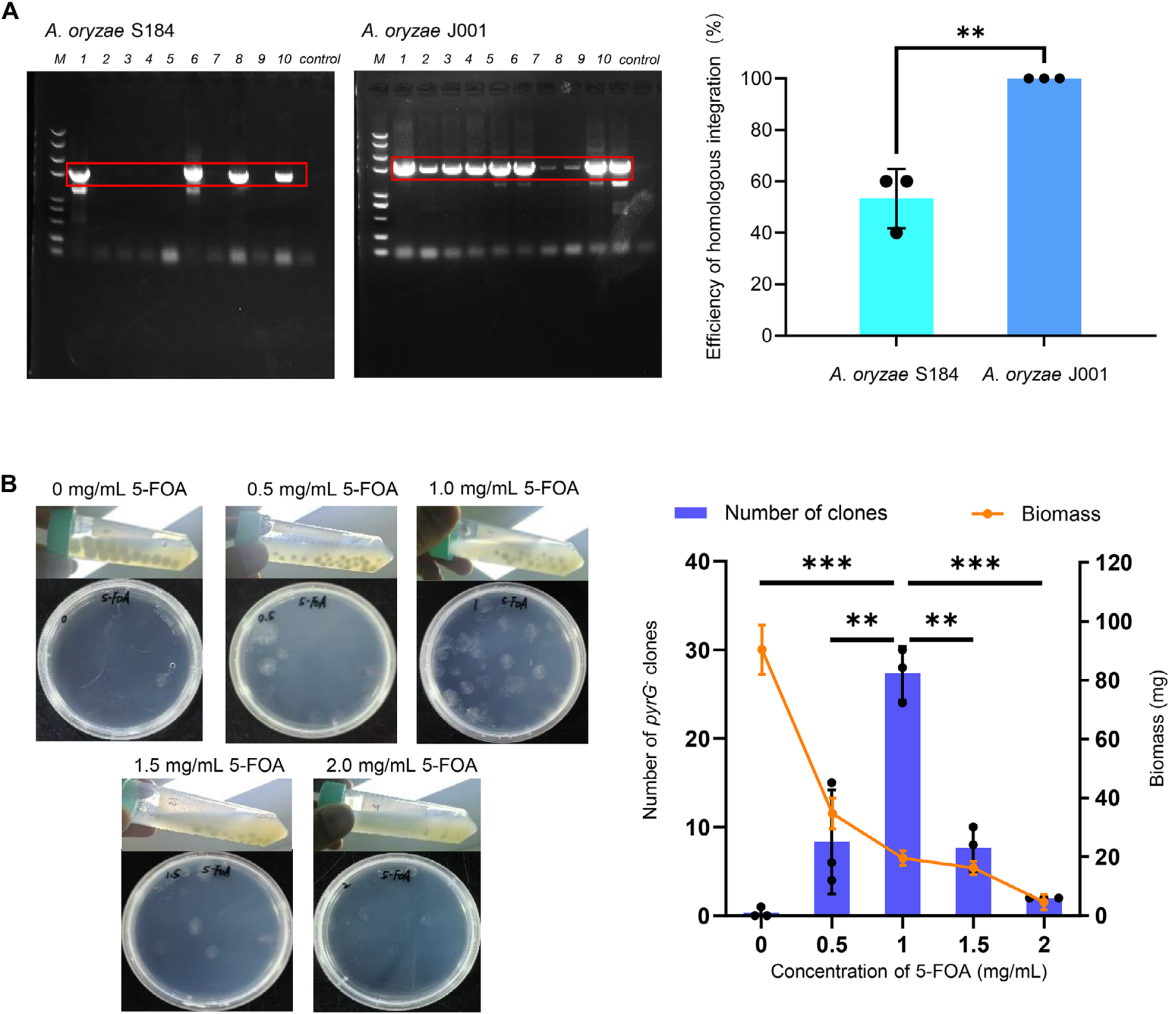
Optimization of the *A. oryzae* heterologous expression system. A) Comparison of the site‐specific integration efficiency between *A. oryzae* S184 and *A. oryzae* J001; B) The refined counter‐selection system using the liquid medium with different concentrations of 5‐FOA. Data are shown as mean values ± SD (n = 3). *p < 0.05, **p < 0.01, ***p < 0.001, and n.s., not significant.

The non‐homologous end joining (NHEJ) pathway in *A. oryzae* can significantly reduce homologous recombination frequency,^[^ ^18^ ^]^ leading to a high incidence of false‐positive clones. To enhance the site‐specific integration of exogenous target genes, we engineered *A. oryzae* S184 by knocking out the *ku80* gene. The Ku70‐Ku80 heterodimer is a key component of the NHEJ pathway, where it functions to recognize and bind to broken DNA ends, subsequently recruiting other repair factors to mediate the ligation of these ends without relying on homologous sequences.^[^ ^19^ ^]^ By deleting *ku80*, we aimed to suppress the error‐prone NHEJ pathway, thereby promoting the more precise homologous recombination pathway and ultimately enhancing the efficiency of target gene integration. A *ku80*‐cutting Cas9 plasmid and a *ku80*‐disrupting donor plasmid were constructed (Figure S2A, Supporting Information), and co‐introduced into *A. oryzae* S184 to generate the *∆ku80* mutant. The successful deletion of *ku80* was validated by PCR analysis (Figure S2B, Supporting Information). The self‐replicating Cas9 plasmid was then removed through counter‐selection, resulting in *A. oryzae* J001, which was designated as the parent strain for gene incorporation. To evaluate the efficiency of site‐specific integration, the *β*‐glucuronidase (*GUS*) gene was introduced into the HS801 locus of both *A. oryzae* S184 and J001.^[^ ^20^ ^]^ Results showed that the *A. oryzae* S184 introduced with Cas9 plasmid alone generated a substantial number of false‐positive clones. In contrast, *A. oryzae* J001 could only grow when both the Cas9 and donor plasmids were added simultaneously (Figure S3, Supporting Information), indicating that false‐positive clones generated by NHEJ were significantly reduced. PCR analysis confirmed that the integration efficiency of the *A. oryzae* S184 strain was approximately 50%, while the successful integration efficiency of the *A. oryzae* J001 reached nearly 100% (**Figure** **1**A).

After the site‐specific integration of targe genes, eliminating the autonomous Cas9 plasmid with the *pyrG* expression cassette is essential for the subsequent recyclable use of the *pyrG* selection marker. The conventional method involves culturing the resulting *pyrG^+^* transformants in non‐selective medium for two to three generations, followed by spreading the transformants on the plates containing uracil and 5‐fluoroorotic acid (5‐FOA) to screen *pyrG^−^* strains.^[^ ^17^, ^21^ ^]^ However, this procedure is time‐consuming (15–18 days) and ineffective. To shorten the time required for selection marker recycling, we explored an alternative approach. Instead of using non‐selective medium, we inoculated the transformants into liquid medium supplemented with different concentrations of 5‐FOA. After the transformants had been cultured for 2 days, 10 µL of the culture broth was sampled and then spread onto the selective medium containing uracil and 5‐FOA, followed by additional 4 days of culture. The results demonstrated that when the *pyrG^+^* transformant was cultured without 5‐FOA, substantial biomass could be accumulated, but few lost the plasmid, and almost no *pyrG^−^* strains were obtained (Figure 1B). When the concentration of 5‐FOA was increased to 1.0 mg L^−1^, the biomass slightly decreased, but *pyrG^−^* strains could be efficiently enriched. Further increasing the 5‐FOA concentration led to significantly reduced biomass and almost no *pyrG^−^* strains. Our optimized procedure only requires culturing the *pyrG^+^* transformants with 1.0 mg L^−1^ 5‐FOA for one generation, reducing the process duration from 15–18 days to only 5–6 days (Figure S4, Supporting Information).

### 2.2 Heterologous Reconstitution of an Artificial Biosynthetic Pathway Potentially Involved in the Formation of Enfumafungin

**Figure 2:**
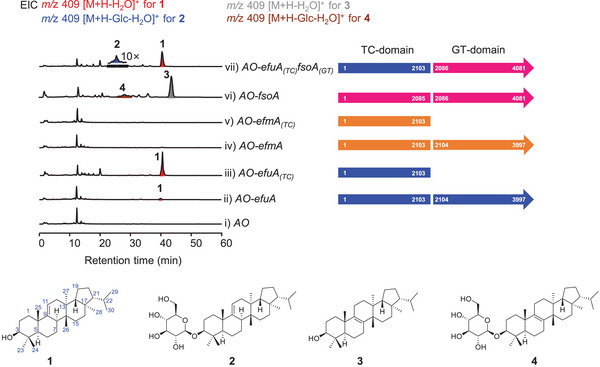
Heterologous expression of the standalone TC domain and TC‐GT fusion enzyme in *A. oryzae*.

To enhance supply of 2,3‐oxidosqualene, we first optimized the mevalonate pathway in *A. oryzae* J001, yielding *A. oryzae* J002. Next, we introduced the native fusion enzyme gene *efuA* into the HS401 locus of *A. oryzae* J002.^[^ ^20^ ^]^ However, the resulting strain *AO‐efuA* produced a low titer of fernenol (**1**) and no detectable glucoside (**2**) (**Figure** **2**, lines i and ii). This is unexpected since the TC domain of EfuA can efficiently catalyze the fernenol formation (Figure 2, line iii). Given that glycosylation is crucial in the biosynthesis of enfumafungin‐type antibiotics,^[^ ^1^ ^]^ we decided to explore alternative analogues of EfuA. We identified a candidate gene cluster (*efm*) in *Hormonema carpetanum* CBS 115712, which shared high amino acid sequence identities to those encoded by the *efu* cluster (Figure S5, Supporting Information). Unfortunately, neither the full‐length EfmA nor its truncated TC domain EfmA_(TC)_ produced any detectable products in *A. oryzae* J002 (Figure 2, lines iv and v; Notes S1 and S2, Supporting Information). However, FsoA can normally produce isomotiol (**3**) and its glycosylated form **4** in *A. oryzae* J002 (Figure 2, line vi). These results implied that the natural fusion enzymes involved in the biosynthesis of enfumafungin cannot be functionally expressed in the heterologous host. Thus, we turned to our pre‐designed fusion enzyme EfuA_(TC)_FsoA_(GT)_, composed of the TC domain of EfuA and the GT domain of FsoA, which functions normally in *A. oryzae* NSAR1.^[^ ^1^ ^]^ As expected, integration of *efuA_(TC)_fsoA_(GT)_* into the HS401 locus of *A. oryzae* J002 led to substantial accumulation of fernenol, along with a small amount of its glycosylated derivative (Figure 2, line vii).

Regarding the impaired catalytic function of EfuA in *A. oryzae* J002, we hypothesized that the fusion of EfuA_(GT)_ to EfuA_(TC)_ may lead to aberrant intron splicing, improper protein folding, or incorrect intracellular localization. Given the host dependence of *efuA* expression, it is also plausible that EfuA could exert its catalytic function properly in alternative heterologous hosts beyond *A. oryzae*.^[^ ^22^ ^]^ To test whether fusion of EfuA_(GT)_ to other TC domains would impair their terpene cyclization activity in *A. oryzae*, we constructed the artificial fusion enzyme FsoA_(TC)_EfuA_(GT)_. Through heterologous expression in *A. oryzae* J002, we found that when fused with EfuA_(GT)_, FsoA_(TC)_ could still produce the desired compound **3** (Figure S6, Supporting Information). This indicates that EfuA_(GT)_ does not universally inhibit all TC domains.

**Figure 3:**
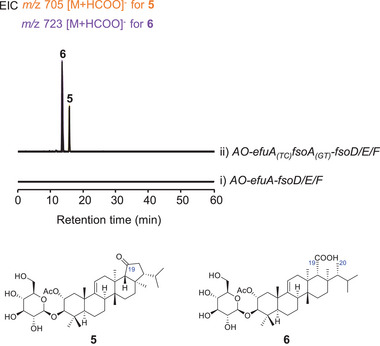
Reconstitution of an artificial biosynthetic pathway potentially involved in the formation of enfumafungin in *A. oryzae* through introduction of the artificial fusion enzyme EfuA_(TC)_FsoA_(GT)_ and FsoD/E/F.

To determine if the artificial fusion enzyme EfuA_(TC)_FsoA_(GT)_ could enable the heterologous biosynthesis of enfumafungin, we introduced the tailoring enzyme genes into the HS601 locus of *AO*‐*efuA_(TC)_fsoA_(GT)_*.^[^ ^20^ ^]^ Given that the uncharacterized EfuH2, EfuH1, and EfuG exhibit high amino acid sequence identities of 55%, 42%, and 56% with FsoD, FsoF, and FsoE, respectively (Figures S5 and S7–S9, Supporting Information), and considering the significant structural similarity between fernenol and isomotiol, we hypothesized that the tailoring enzymes from the fuscoatroside biosynthetic pathway could serve as functional analogs. When *fsoD*, *fsoE* and *fsoF* were introduced, the resulting strain *AO‐efuA_(TC)_fsoA_(GT)_‐fsoD/E/F* produced two additional products **5** and **6**, while the strain *AO‐efuA‐fsoD/E/F* did not (**Figure** **3**). Through large‐scale fermentation, **5** and **6** were isolated (Note S3, Supporting Information), and subsequently subjected to unambiguous structural characterization. Both **5** and **6** are characterized by the presence of a 2‐acetoxyl group and a glucosyloxy group at C3. The difference lies in the E‐ring: **6** exhibits a cleaved E‐ring, while **5** possesses an intact E‐ring with a 19‐keto group. These characteristics indicate that **5** and **6** are putative intermediates in enfumafungin biosynthesis. Consequently, we achieved the reconstitution of an artificial biosynthetic pathway potentially involved in the formation of enfumafungin in *A. oryzae* by combining the artificial enzyme EfuA_(TC)_FsoA_(GT)_ with the three tailoring enzymes FsoD/E/F from fuscoatroside pathway.

**Figure 4:**
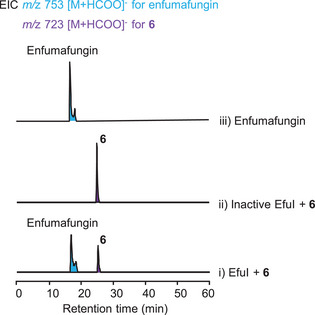
In vitro enzymatic assay of EfuI with addition of **6**.

As for the characteristic hemiacetal moiety between C24 and C25, the remaining P450 enzyme gene *efuB* and/or the Fe(II)/α‐KG‐dependent oxygenase gene *efuI* in the *efu* cluster (Figure S5, Supporting Information) likely participated in its formation. Due to its solubility, we firstly prepared the recombinant EfuI by expressing the intron‐free *efuI* in *Escherichia coli* (Figure S10A, Supporting Information). Notably, upon incubation of EfuI with **6** and the cofactor α‐ketoglutaric acid, the formation of enfumafungin was observed (Figures 4; S10B, Supporting Information), suggesting that EfuI serves as a multifunctional Fe(II)/α‐KG‐dependent oxygenase capable of catalyzing a six‐electron oxidation to yield the hemiacetal structure. Additionally, in vitro enzymatic assays showed that EfuI is probably able to catalyze the oxidation of C24 and C25 in fuscoatroside to give the hemiacetal moiety (Figure S10C and D, Supporting Information), implying its promiscuity toward the double bond position of the skeleton. However, when *efuI* was introduced into *AO‐efuA_(TC)_fsoA_(GT)_‐fsoD/E/F*, only trace amounts of enfumafungin were detected with almost no consumption of **6** (Figure S11, Supporting Information). We hypothesized that the potent antifungal activity of enfumafungin is detrimental to *A. oryzae*, thereby inhibiting its biosynthesis. Alternatively, as all the enzymes introduced into *A. oryzae*, except for EfuI, are not the native enzymes responsible for biosynthesis of enfumafungin, we could not exclude the possibility that some differences between the non‐native and native enzymes may contribute to weakened biosynthesis of enfumafungin. In the future, introduction of the self‐resistance gene into the host or utilization of the native tailoring enzymes might potentially overcome the obstacle.

### 2.3 Elucidation of the Sequence of Tailoring Reactions

**Figure 5:**
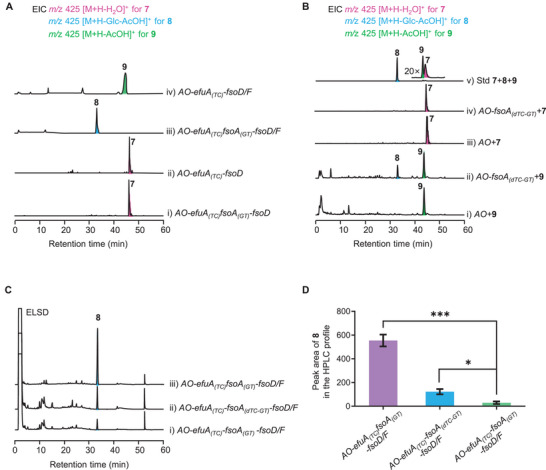
In‐depth analysis of the catalytic function and activity of the GT domain. A) Investigation of the catalytic sequence of FsoD, FsoF, and the GT domain through stepwise introduction of tailoring enzyme genes; B) Confirmation of the function of the GT domain through feeding experiments; C) Comparative analysis of the catalytic activity of the GT domain in its standalone form versus its fusion with the TC domain through heterologous expression; D) The peak area of **8** in the HPLC profile of crude extracts obtained from *A. oryzae* transformants possessing the single GT domain or the intact TC‐GT fusion enzyme. Data are shown as mean values ± SD (n = 3). *p < 0.05, **p < 0.01, ***p < 0.001, and n.s., not significant.

**Figure 6:**
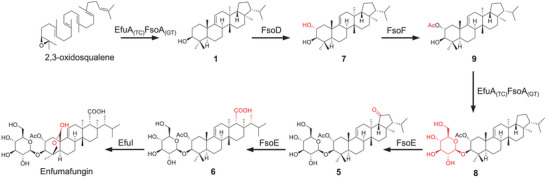
The artificial biosynthetic pathway of enfumafungin reconstructed in *A. oryzae*.

To clarify the reaction sequence of the artificial biosynthetic pathway in *A. oryzae* J002, the tailoring enzyme genes were step by step introduced into the host. Based on the isolation of **5** and **6**, we proposed that acetoxylation and glycosylation occur prior to the cleavage of E‐ring. Therefore, *fsoD* was first introduced into *AO‐efuA_(TC)_fsoA_(GT)_*, and only one additional product **7** was detected in the strain *AO‐efuA_(TC)_fsoA_(GT)_‐fsoD* (**Figure** **5**A, line i), which was elucidated as the C2 hydroxylated derivative of fernenol. The result suggested that fernenol, released from the TC domain of EfuA_(TC)_FsoA_(GT)_, was directly captured by FsoD rather than the spatially proximal GT domain. To further validate the assumption, *fsoD* was also transformed into *AO‐efuA_(TC)_*, and **7** was indeed observed in *AO‐efuA_(TC)_‐fsoD* (Figure 5A, line ii). Subsequently, with addition of *fsoF*, we found that *AO‐efuA_(TC)_fsoA_(GT)_‐fsoD/F* significantly accumulated a new product **8** (Figure 5A, line iii), which is produced by acetylation and glycosylation of **7** (**Figure** **6**). Given that the glucoside of **7** was not detected in *AO‐efuA_(TC)_fsoA_(GT)_‐fsoD*, we thus proposed that **7** is firstly subjected to acetylation, followed by glycosylation to turn into **8**. To test whether **7** could be acetylated by FsoF without requirement of glycosylation, we constructed the strain *AO‐efuA_(TC)_‐fsoD/F*. In accordance with our proposal, the acetylated form **9** of **7** was detected (Figure 5A, line iv). To investigate whether acetylation of 2‐OH is a prerequisite of 3‐OH glycosylation, feeding experiments were carried out. We prepared the mutated variant *fsoA_(dTC‐GT)_* of *fsoA* through site‐directed mutagenesis, which can encode the full‐length fusion protein with the inactive TC domain, and then introduced *fsoA_(dTC‐GT)_* into *A. oryzae* J001. With individual addition of **7** and **9**, we found that *AO‐fsoA_(dTC‐GT)_* could convert **9** to the corresponding glucoside **8**, but not **7** (Figure 5B), confirming the necessity of 2‐OH acetylation for 3‐OH glycosylation. These results demonstrated that despite the fusion of the TC and GT domains in EfuA_(TC)_FsoA_(GT)_, this enzyme catalyzes non‐consecutive terpene cyclization and glycosylation.

To determine whether the biosynthesis of fuscoatroside follows the same sequence, we introduced the *fsoA* (or *fsoA_(TC)_*) and *fsoD/F* genes into the *A. oryzae* J002. Both *AO‐fsoA‐fsoD* and *AO‐fsoA_(TC)_‐fsoD* could only produce the C2 hydroxylated derivative **10** of **3**. *AO‐fsoA_(TC)_‐fsoD/F* could yield a putative 2‐OH acetylated product **11** of **10**, while *AO‐fsoA‐fsoD/F* could not only produce **11** but also its glycosylated product **12** (Figure S12, Supporting Information). These results suggested that FsoA_(GT)_ also requires an acetylated substrate for glycosylation during the biosynthesis of fuscoatroside. This indicated that the two domains of the fusion enzymes involved in the biosynthesis of enfumafungin‐type antibiotics catalyze non‐consecutive reactions, which differs from the activity of canonical fusion enzymes.

In general, fusion enzymes predominantly catalyze sequential reactions.^[^ ^23^, ^24^ ^]^ Given that the TC‐GT fusion enzymes responsible for enfumafungin‐type antibiotics biosynthesis catalyze non‐consecutive cyclization and glycosylation, it is confusing why the TC and GT domains are linked together. To obtain further insight into the atypical fusion enzyme, we planned to undertake the comparative analysis of the catalytic activity between the intact fusion enzymes and the excised domains through heterologous expression. To this end, *fsoA_(GT)_*, which encodes the single GT domain, and *fsoA_(dTC‐GT)_* were introduced into *AO‐efuA_(TC)_‐fsoD/F*, respectively. We found that *AO‐efuA_(TC)_‐fsoD/F‐fsoA_(GT)_* produced a lower titer of **8** than *AO‐efuA_(TC)_‐fsoD/F‐fsoA_(dTC‐GT)_* and *AO‐efuA_(TC)_fsoA_(GT)_‐fsoD/F* (Figure 5C and D), indicating that the standalone GT domain is catalytically active, but its activity is weaker than that of the full‐length fusion proteins. We hypothesized that the fusion of the TC and GT domains may facilitate co‐translational folding of the multidomain protein, a process that promotes proper domain‐by‐domain folding and minimizes misfolding.^[^ ^25^ ^]^ This could explain why the two domains remain fused despite their non‐consecutive roles in biosynthetic transformations.

From an evolutionary perspective, over millions of years, the genes of initially independent enzymes, which are involved in a sequential reaction, may tend to join together to encode fusion enzymes, driven by the need to enhance the substrate transfer efficiency.^[^ ^26^ ^]^ The majority of naturally occurring fusion enzymes thereby catalyze consecutive reactions. For instance, multi‐domain polyketide synthases sequentially catalyze a series of reactions to convert one starter unit and several extender units to polyketides,^[^ ^27^ ^]^ and fungi‐derived bifunctional sesterterpene synthases catalyze formation of geranylfarnesyl pyrophosphate and subsequent cyclization.^[^ ^28^ ^]^ However, several exceptions have also been reported.^[^ ^29^ ^]^ Taking the fusion protein HS‐HMGS as an example, it consists of a sesterterpene hirsutene synthase domain and a 3‐hydroxy‐3‐methylglutaryl‐CoA (HMG‐CoA) synthase domain, and catalyzes formation of HMG‐CoA and cyclization of geranylgeranyl pyrophosphate to hirsutene.^[^ ^30^ ^]^ The TC‐GT fusion enzymes involved in biosynthesis of enfumafungin‐type antibiotics provide an additional example that fusion proteins catalyze non‐consecutive reactions. Among these uncommon fusion enzymes, several were reported to possess no apparent functional advantages compared to their independent domains.^[^ ^30^, ^31^ ^]^ In contrast, the TC‐GT fusion enzymes exhibit significantly higher catalytic activity than the separated domains. We thus inferred that the fusion of the TC and GT domains during evolution is not a serendipitous event, but may be driven by the force aimed at improving the catalytic activity.

**Figure 7:**
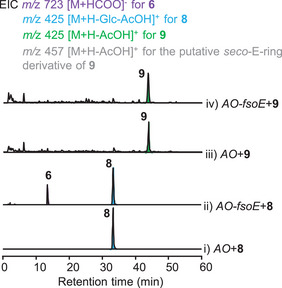
Confirmation of the function of FsoE through feeding experiments.

Finally, to determine whether FsoE is able to utilize the glycoside **8** as the substrate to give **6**, or convert the aglycone **9** to the corresponding *seco*‐E‐ring product, we constructed *AO‐fsoE*, and then fed **8** and **9** to the strain, respectively. The results showed that *AO‐fsoE* can only convert **8** to **6** (**Figure** **7**), confirming that glycosylation of 3‐OH is essential for the FsoE‐catalyzed cleavage of the C19 and C20 single bond (Figure 6).

## 3 Conclusion

Through disruption of the *ku80* gene in *A. oryzae* S184 and refinement of the counter‐selection procedure, the *A. oryzae* J001 expression system was established with the increased site‐specific integration frequency and shortened time for removing the *pyrG* selection marker. Subsequently, the artificial biosynthetic pathway potentially involved in biosynthesis of enfumafungin was successfully reconstructed in the *A. oryzae* host. Notably, the natural TC‐GT fusion enzyme involved in enfumafungin biosynthesis almost could not be functionally expressed in *A. oryzae*. Nevertheless, the pre‐designed fusion enzyme EfuA_(TC)_FsoA_(GT)_, along with fsoD/E/F involved in fuscoatroside biosynthetic pathway, could effectively produce the putative enfumafungin intermediates. Moreover, we found that despite the significance of fusion of the TC and GT domains for their catalytic activity, the TC‐GT fusion enzymes for enfumafungin‐type antibiotics biosynthesis catalyze non‐consecutive reactions, different from typical fusion enzymes. Specifically, the GT domain cannot catalyze glycosylation until the fernane skeleton generated by the TC domain is installed with the acetoxyl group at the C2 position.

## 4 Experimental Section

### General Materials and Experimental Procedures

Synthesis of genes and primers were performed by Sangon Biotech Co., Ltd. (Shanghai, China) or Tsingke Biotech Co., Ltd. (Beijing, China). PCR was conducted using an A100 thermal cycler (LongGene, China) and 2×Phanta Flash Master Mix (Vazyme, China). FastDigest enzymes (Thermo Scientific, USA) were used for linearization of the plasmid. Ligation of the DNA fragment with the digested plasmid was accomplished using the ClonExpress II One Step Cloning Kit (Vazyme, China) or the ClonExpress Multis One Step Cloning Kit. The FastPure Plasmid Mini Kit (Vazyme, China) was used for plasmid extraction. Silica gel (200–300 mesh) from Qingdao Haiyang Chemical Co., Ltd. (Qingdao, China) and ODS (50 µm) from YMC Co., Ltd. (Kyoto, Japan) were used for the column chromatography. Pyridine‐*d*_5_ and CDCl_3_ were purchased from Cambridge Isotope Laboratories, Inc. (Massachusetts, USA).

High‐performance liquid chromatography‐mass spectrometry (HPLC‐MS) analysis was carried out using a Dionex UltiMate 3000 HPLC system (Thermo Scientific, USA) and an amaZon SL ion trap mass spectrometer coupled with an atmospheric pressure chemical ionization (APCI) source (Bruker, USA). The semi preparative HPLC was performed on a Dionex UltiMate 3000 HPLC system. The medium‐pressure liquid chromatography (MPLC) was performed on a Cheetah Pro System (Tianjin Agela Technologies Co., Ltd, China). The optical rotation values were recorded on a JASCO P1020 digital polarimeter from JASCO International Co., Ltd. (Tokyo, Japan). Bruker AV 400/600 spectrometers (USA) were employed to measure 1D and 2D NMR spectra using the solvent signals (pyridine‐*d*_5_: *δ*_H_ 7.21/*δ*_C_ 123.5; CDCl_3_: *δ*_H_ 7.26/*δ*_C_ 77.0) as internal standards.

### Strains and Media

*Hormonema carpetanum* CBS 115712 was obtained from Centraalbureau voor Schimmelcultures (CBS) and cultured in the PDB medium for extraction of genomic DNA. The *pyrG*‐deficient *Aspergillus oryzae* S184^[^ ^17^ ^]^ serves as the parent strain for subsequent genetic modifications. *Escherichia coli* DH5α (Takara, Japan) for construction of recombinant plasmids was cultured in LB medium with 100 mg L^−1^ ampicillin.

### Construction of Recombinant Plasmids

To construct the plasmid that was used for CRISPR/Cas9‐based cleavage of DNA, the guide RNA was designed by CRISPOR online platform (https://crispor.gi.ucsc.edu/crispor.py), and then the synthesized oligonucleotide fragment was ligated with the U6 promoter/terminator cassette via overlap PCR. The resulting gRNA expression cassette was then inserted into the NotI linearized pSC‐134 plasmid,^[^ ^17^ ^]^ which harbors the *Cas9* expression cassette, the *pyrG* expression cassette and the AMA1 replicator.

To construct the plasmid serving as the donor for integration of the exogenous gene via homologous recombination, the pESC‐Ura plasmid was utilized to construct the tool plasmids containing 1300 bp homologous arms. The cloning site, which features the SmaI recognition sequence, was embedded between the upstream and downstream homologous arms. For targeted gene integration, the exogenous gene was introduced into pTAex3 or pUSA, which comprise the *amyB* promoter/terminator cassette. Subsequently, the exogenous gene expression cassettes amplified from the pTAex3 or pUSA‐based recombinant plasmids were ligated with SmaI‐digested donor plasmids. All the primers and plasmids were listed in Tables S1 and S2 (Supporting Information).

### Transformation of Aspergillus oryzae

*A. oryzae* transformants were generated via PEG‐mediated transformation of protoplasts. The parent strain was grown in DPY medium (1% polypeptone, 2% dextrin, 0.5% yeast extract, 0.5% KH_2_PO_4_, 0.05% MgSO_4_·7H_2_O) with supplement of 0.2% uracil for 1 day, and then the harvested mycelia were digested using the Yatalase solution (1% Yatalase, 0.6 M (NH_4_)_2_SO_4_, 50 mM maleic acid, pH 5.5) at 30 °C for 3 h to remove cell walls. After centrifugation at 1500 rpm for 10 min, the protoplasts were obtained, followed by being washed twice with TF Solution 2 (1.2 M sorbitol, 35 mM NaCl, 50 mM CaCl_2_·2H_2_O, 10 mM Tris‐HCl, pH 7.5). The protoplasts were resuspended in TF Solution 2 to a concentration of approximately 1.0 × 10^7^ cells mL^−1^. Subsequently, 200 µL of protoplast suspension was incubated with 10–30 µL of recombinant plasmids (≈1000 ng µL^−1^) on ice for 30 min. With addition of 1.35 mL TF Solution 3 (60% PEG4000, 50 mM CaCl_2_·2H_2_O, 10 mM Tris‐HCl, pH 7.5) in three times, the mixture was incubated at the room temperature for 20 min followed by addition of 5 mL TF solution 2. After centrifugation at 1500 g for 10 min, the supernatant was discarded, and the precipitate was resuspended in 400 µL TF solution 2. 200 µL of the mixture was plated onto the selective medium (0.2% NH_4_Cl, 0.1% (NH_4_)_2_SO_4_, 0.05% KCl, 0.15% KH_2_PO_4_, 0.05% MgSO_4_·7H_2_O, 2% glucose, 21.8% sorbitol, 0.15% methionine, 0.1% arginine and 0.01% adenine, 2% agar), and then covered with the selective medium. The transformants could be obtained after incubation at 30 °C for 3–4 days. All the transformants used in this work were listed in Table S3 (Supporting Information).

### Elimination of the Self‐Replicating Plasmid in Aspergillus oryzae Transformants

*A. oryzae* transformants were cultivated in the 10 mL DPY medium (1% polypeptone, 2% dextrin, 0.5% yeast extract, 0.5% KH_2_PO_4_, 0.05% MgSO_4_·7H_2_O) supplemented with 0.2% uracil and 0.1% 5‐FOA for 2–3 days to facilitate the discarding of the self‐replicating plasmid. Subsequently, the culture broth was diluted by one hundred times, and 1 mL of the diluent was spread on the counter‐selection medium (0.2% NH_4_Cl, 0.1% (NH_4_)_2_SO_4_, 0.05% KCl, 0.15% KH_2_PO_4_, 0.05% MgSO_4_·7H_2_O, 2% glucose, 21.8% sorbitol, 0.15% methionine, 0.1% arginine, 0.01% adenine, 0.2% uracil, 0.13% 5‐fluoroorotic acid, 2% agar). After being incubated at 30 °C for 3 days, the *pyrG^−^* phenotype strains were obtained, which were further validated through individual transferring onto the DPY agar plate with or without 0.2% uracil.

### Extraction and Analysis of Metabolites

After *A. oryzae* transformants growing in the modified Czapek‐Dox (CD) medium (1% polypeptone, 2% starch, 0.3% NaNO_3_, 0.2% KCl, 0.1% KH_2_PO_4_, 0.05% MgSO_4_·7H_2_O, 0.002% FeSO_4_·7H_2_O, pH 5.5) for 5 days, mycelia were extracted with ethanol, and culture broth was extracted with ethyl acetate (EtOAc). For HPLC analysis, the mobile phase was composed of H_2_O with 0.1% formic acid (A) and CH_3_CN with 0.1% formic acid (B), and a COSMOSIL 3C18‐EB Column (4.6 mm i.d. × 150 mm, 5 µM) was used. For analysis of metabolites containing compounds **1**–**4**, the samples were subjected to a linear gradient elution of 50–100% B (0–10 min) and 100% B (10–50 min) at a flow rate of 1 mL min^−1^. Whereas, for analysis of metabolites containing compounds **5**–**12**, a linear gradient elution of 50–100% B (0–30 min) and 100% B (30–45 min) was utilized. For analysis of metabolites containing enfumafungin, a linear gradient elution of 30–100% B (0–40 min) and 100% B (40–55 min) was employed.

### Feeding Experiments

The *A. oryzae* transformant was inoculated to 10 mL DPY medium for 2–3 days, and then transferred into 100 mL modified CD medium to induce the gene expression. After 24 hours, 100 µg of the substrate dissolved in 200 µL DMSO were added to the culture broth, and then the transformant was further cultivated for 3 days. Subsequently, the mycelia were extracted with ethanol and the broth was extracted with EtOAc. The resulting extract was subjected to HPLC‐MS analysis.

### Expression and Purification of the Recombinant EfuI

*Escherichia coli* Rosetta (DE3) containing the pET28b‐*efuI* plasmid was cultured in LB medium supplemented with 50 mg L^−1^ kanamycin sulfate at 37 °C and 220 rpm for 16 h. When the OD_600_ reached 0.6, protein expression was induced by addition of 0.4 mM isopropyl *β*‐d‐thiogalactoside, followed by further incubation at 16 °C and 160 rpm for 20 h. The cells were harvested through centrifugation at 4500 g for 30 min and resuspended in 50 mM Tris‐HCl buffer (pH 7.5). After sonication on ice, the mixture was centrifuged at 12000 g for 30 min, and the supernatant was purified by Ni‐NTA affinity chromatography (Qiagen). The protein solution was concentrated using Amicon Ultra‐15 centrifugal filter column (50 K MWCO, Millipore). The purified enzyme was analyzed by sodium dodecyl sulfate‐polyacrylamide gel electrophoresis (SDS‐PAGE), and its concentration was determined by measuring the absorbance at 280 nm.

### In Vitro Enzymatic Assay of EfuI

The reaction mixture, with a total volume of 500 µL, consisted of 50 mM Tris‐HCl (pH 7.5), 0.64 mM α‐ketoglutaric acid, 1 mM of sodium ascorbate, 0.2 mM FeSO_4_·7H_2_O, 0.4 mM substrate and 1.3 µM EfuI. After incubation at 30 °C for 12 h, the mixture was extracted twice with EtOAc and then concentrated for HPLC‐MS analysis.

### Statistical Analysis

GraphPad Prism 10 was used for statistical analyses. *p*‐values were calculated with unpaired t‐test method. Significance was determined as *p < 0.05, **p < 0.01, ***p < 0.001, and n.s., not significant. The experiments were repeated independently three times.

### Structural Characterization

(2*α*,3*β*)‐2‐Acetyloxy‐3‐(*β*‐d‐glucopyranosyloxy)‐fern‐9(11)‐en‐19‐one (**5**) A white powder; [*α*]25 D = −43.7 (*c* 0.11, C_5_H_5_N). NMR spectra, see Figure S13 (Supporting Information); NMR data, see Table S4 (Supporting Information).

∆^9(11)^‐Iso‐fuscoatroside (**6**) A white powder; [*α*]25 D = −80.3 (*c* 0.3, C_5_H_5_N). NMR spectra, see Figure S14 (Supporting Information); NMR data, see Table S5 (Supporting Information).

Fern‐9(11)‐ene‐2*α*,3*β*‐diol (**7**) A white powder; [*α*]25 D = ‐64.2 (*c* 0.25, CHCl_3_). NMR spectra, see Figure S15 (Supporting Information). NMR data, see Table S6 (Supporting Information). The NMR data were in good accordance with those reported.^[^ ^32^, ^33^ ^]^

(2*α*,3*β*)‐2‐Acetyloxy‐fern‐9(11)‐en‐3‐yl *β*‐d‐glucopyranoside (**8**) A white powder; [*α*]25 D = −11.6 (*c* 0.12, CHCl_3_). NMR spectra, see Figure S16 (Supporting Information); NMR data, see Table S7 (Supporting Information).

2*α*‐Acetyloxy‐fern‐9(11)‐en‐3*β*‐ol (**9**) A white powder; [*α*]25 D = −64.8 (*c* 0.11, CHCl_3_). NMR spectra, see Figure S17 (Supporting Information); NMR data, see Table S8 (Supporting Information).

## Conflict of Interest

The authors declare no conflict of interest.

## Supporting information



Supporting Information

## Data Availability

The data that support the findings of this study are available from the corresponding author upon reasonable request.
