# Supplementary material for: Engineered Fusion Enzyme‐Mediated Non‐Consecutive Cyclization‐Glycosylation Enables Heterologous Synthesis of Antifungal Enfumafungin
Source: Adv Sci (Weinh). 2025 Sep 2;12(44):e07531. doi: 10.1002/advs.202507531 (PMC12667498; doi:10.1002/advs.202507531)
Supplement: Supplementary file 1 — Supporting Information [file ADVS-12-e07531-s001.doc]

Supporting Information

Engineered Fusion Enzyme-Mediated Non-Consecutive Cyclization-Glycosylation Enables Heterologous Synthesis of Antifungal Enfumafungin

*Yaohui Gao****^⊥^****, Jianming Lv****^⊥^****, Yue Zhong****^⊥^****, Zhiqin Cao, Rui Luo, Yue Qi, Gaoqian Wang, Shaoyang Li, Guodong Chen, Dan Hu*, Hao Gao*, Xinsheng Yao**

*Corresponding authors: [tyaoxs@jnu.edu.cn](mailto:tyaoxs@jnu.edu.cn) (Xinsheng Yao), tghao@jnu.edu.cn (Hao Gao), [thudan@jnu.edu.cn](mailto:thudan@jnu.edu.cn) (Dan Hu).

**^⊥^**These authors contributed equally to this work.

**Table of Contents**

[Supplementary Notes 2](#__RefHeading___Toc205243197)

[Note S1. **The nucleotide sequence of *efmA_(TC)_*** 2](#__RefHeading___Toc205243198)

[Note S2. **The nucleotide sequence of *efmA*** 4](#__RefHeading___Toc205243199)

[Note S3. **Isolation and purification of metabolites** 6](#__RefHeading___Toc205243200)

[Supplementary Tables 7](#__RefHeading___Toc205243201)

[Table S1. **Primers used in the study** 7](#__RefHeading___Toc205243202)

[Table S2. **Plasmids used in the study** 10](#__RefHeading___Toc205243203)

[Table S3. **Strains used in the study** 12](#__RefHeading___Toc205243204)

[Table S4. **NMR assignments for** 5 **(^1^H for 600 MHz and ^13^C for 150 MHz in pyridine-*d*_5_)** 13](#__RefHeading___Toc205243205)

[Table S5. **NMR assignments for** 6 **(^1^H for 600 MHz and ^13^C for 150 MHz in pyridine-*d_5_*)** 14](#__RefHeading___Toc205243206)

[Table S6. **NMR assignments for** 7 **(^1^H for 600 MHz and ^13^C for 150 MHz in CDCl_3_)** 15](#__RefHeading___Toc205243207)

[Table S7. **NMR assignments for** 8 **(^1^H for 400 MHz and ^13^C for 100 MHz in pyridine-*d*_5_)** 16](#__RefHeading___Toc205243208)

[Table S8. **NMR assignments for** 9 **(^1^H for 600 MHz and ^13^C for 150 MHz in pyridine-*d_5_*)** 17](#__RefHeading___Toc205243209)

[Supplementary Figures 18](#__RefHeading___Toc205243210)

[Figure S1. **Representative enfumafungin-type antibiotics.** 18](#__RefHeading___Toc205243211)

[Figure S2. **Construction of the *∆ku80* mutant *A. oryzae* J001.** 19](#__RefHeading___Toc205243212)

[Figure S3. **Comparison of the site-specific integration efficiency of *A. oryzae* S184 and *A. oryzae* J001.** 20](#__RefHeading___Toc205243213)

[Figure S4. **Scheme of the counter-selection process to eliminate the *pyrG* selection marker.** 21](#__RefHeading___Toc205243214)

[Figure S5. **Amino acid sequence identities of the proteins encoded by the gene clusters *fso*, *efu*, and *efm*.** 22](#__RefHeading___Toc205243215)

[Figure S6. **Functional analysis of the fusion enzyme FsoA_(TC)_EfuA_(GT)_.** 23](#__RefHeading___Toc205243216)

[Figure S7. **Amino acid sequence alignment between EfuH2 and FsoD.** 24](#__RefHeading___Toc205243217)

[Figure S8. **Amino acid sequence alignment between EfuH1 and FsoF.** 25](#__RefHeading___Toc205243218)

[Figure S9. **Amino acid sequence alignment between EfuG and FsoE.** 26](#__RefHeading___Toc205243219)

[Figure S10. **In vitro enzymatic assay of EfuI.** 27](#__RefHeading___Toc205243220)

[Figure S11. **Reconstitution of enfumafungin biosynthesis in *A. oryzae* through combination of *efuA_(TC)_fsoA_(GT)_*, *fsoD*, *fsoE*, *fsoF*, and *efuI*.** 28](#__RefHeading___Toc205243221)

[Figure S12. **Elucidation of the catalytic sequence of FsoA_(GT)_ in the biosynthesis of fuscotraside via heterologous expression in *A. oryzae.*** 29](#__RefHeading___Toc205243222)

[Figure S13. **NMR spectra of** 5**.** 32](#__RefHeading___Toc205243223)

[Figure S14. **NMR spectra of** 6**.** 35](#__RefHeading___Toc205243224)

[Figure S15. **NMR spectra of** 7**.** 38](#__RefHeading___Toc205243225)

[Figure S16. **NMR spectra of** 8**.** 41](#__RefHeading___Toc205243226)

[Figure S17. **NMR spectra of** 9**.** 44](#__RefHeading___Toc205243227)

[Reference 45](#__RefHeading___Toc205243228)

Supplementary Notes

Note S1. The nucleotide sequence of *efmA_(TC)_*

ATGGACATGGCGCCAGACGAGTTGGATGAGCTCAGGGGCAGCGCCCAGCGGGCACTTGAGCAGGCCATCGACTTCTCTTTCAGCTGCCAGCAAGATGACGGACACTGGGTGGCGCCCGTCTCAGCCGACGCCACATTCACAGCTCAGTATGTAATGTTCAAGCACGCCATTCCAGCTCTCAACCTGGACATCAGCGGAGCCGAGGCAGCGGCCCTCCGTCACTGGCTTCTCGGAGACCAAAATGCGGCTGAAGGCTCTTGGGGTCTTGCTCCTGGGCTACCGGGAAACTTGTCTACCACAGTCGAGGCATACCTCGCCCTTCGACTCCTCGGTGTACCATCGTCGAACCCAGCACTGCAACAAGCACGCCGTTTTGTGCTGGCTCATGGTGGAATCTCTCGGGTTCGATTTTTTACACGGTTCTTCCTCGCAACCTTTGGCCTGTTTCCGTGGAGCGCCATCCCTCAGATGCCAGCCGAGCTTATACTCATGCCGAAATGGGCTCCCCTGAATATCTACGTCCTCTCATCGTGGGCACGCAGCACCCTGATCCCCATCCTGGTGGTCCGTCATCACGAGCCCCTGTACCCCCTGCCAAATGCCCAAAGCGACCCAAACAGCGGCTTTCTGGACGAACTATGGCTTGACCCAACAAACAAGGAGGTGCCGTTTGCACCGCCGTTGTGGGACATGTTTCATGGGAGAGACCGCGATGTCGTCAAGCTCGCTTTCACTTTGGGGGACAAGGCCCTTGCGCAAATTGGGGGCCTGAAAAAAGGGCCCCAGCGTCGACTTGCCCTTCGGCGTTGCATTGAGTGGCTGCTAGAGCATCAGGAAGAGACGGGCGACTGGGCTGGCTTTTTCCCGCCCATGCACGGCAGTGTCTGGGCGTTGCTTCTTGAAGGCTTTTCTTTGGAACACGACGTCGTGAAGCGGGGACTCGAGGCACTCGAGCGTCTGGCTGTCAACGACGAGAGCGGCAAGTGGCTGCAATCCACAGTGTCGCCGTGCTGGGACACTGCGCTCATGGTCAAGGCCCTTTGCGACGCTGGCCTTGGGCTCGGGGGAGCAGAGGCAGCGAAGGGAAACCGTCATGCGCGGGTCACGACCGCCGTTGATTGGGTCCGCTCACTACAACTCCTCGGCCCTCAGGGTGACTGGCGGGTTTACAGCCGCAACCAGCGCCCGGGGGGCTGGAGTTTCGAGTACAACAACACCTGGTACCCAGATGTGGATGACACGGCGGTTGTGGTCATGATGCTTGTGACGCACGATCCGGCTGCTGTCGAGTCCAATGCCGTTGAAATGGGAATCGAGTGGATTCTTGGGATGCAAAACCACGACGGCGGATGGGGTGCGTTCGACACCAACAACGACGCGCTCTGGCTGCACAAGATTCCTTTCAGCGACATGGACAGCCTCGTCGACCCCAGCACGTCTGATGTGACGGGTCGGATGCTGGAGTGTTTTGGGATGCTCTTGACGCACAGAAAAGGAGGCCTTCGCCTCCGCCCCGAGCTTTCTCAGCGTTTGCATGAATCGGCGCAAAAGGCGTTGGCGTTTCTGTTCAGGGAACAGACGGCCTCAGGTGCGTGGTGGGGACGCTGGGGCTGCAACTACAACTATGGCACAACCAACGTACTCCGCGGTTTGCCCGCTTTCTGCGGAGATAAGGAGGTAGCCAGGGCGGCTCTGCGCGCGGTTCTCTGGCTCGAGAAGTGCCAGAACAAGGATGGCGGTTGGGGCGAGACGTTGCTGTCGTACGGCCACCCAGATCTGGCTGGAAAAGGTCCCAGCACGGCCGCACACACCGCATGGGCGCTGGATGCATTGCTCCGTTTCCGCCCAGCATCCGATCCAGCCTTGCAGAAAGGCGTCCAGTGGCTTGTATCGAATCAAGTTCCCAAGACAGAAGAGAAACGCCATTGGGCCTCATGGCCCTCGGACTTGTATGTTGGCACTGGCTTCCCGAATGTGCTCTACCTTGGATACCCGTTTTACCACCACCACTTTGCCATCTCGGCACTCGCACGGTTTCTCGACAGGACCGACGAACCGGATCAGGACCGTGATCTGCCGCTCCTCATGACTCGGCACGTTTGA

Note S2. The nucleotide sequence of *efmA*

ATGCCATCTTACCACAACACGGACAAGACACTTTTGGGTGATGCTCGTCAATCTCTACAGCAGGCTGTTGACTATTCGCTAGGATGCCAACAAACCGATGGTCACTGGGTGGCTCCAGTTATGGCAGATGCAACCTTTACTGCACAGTATGTCTTTTTCAAGCACCAAATCCCGGAACTCTCATTGGACGAGGATGGCCCCGAGATCCAGAGGTGGCTTTTGGGCGGGCAAACAACAGACGGCTCATGGACTCTTGCGCCTGACCTACCAGGCAATCTGTCTACGACCGTCGAAGCTTACCTTGCGTTACGCATACTTGGTGTTCCTAAGTCCGACCAAGCAATGTTACGAGCACGAGATTTTGTGATCCGTAATGGTGGTGTAGAAGGTGTGCGTTTTTTCACACGCTTCTTCCTTGCGACTTTCGGCCTGGTTCCCTGGACTGCTATCCCGCAGATGCCTGCCGAATTGATATTGTTACCGACCTTCATGTTCTTGAACATCTATGTGCTGTCATCATGGGCACGGAGCACTTTAATTCCTATTCTGTTGGTCCGGCATCACGAACCAGTATATGCCTTGCCCAATGGGCAAAGCGCTCATAACGACTTCCTGGACGAGCTTTGGTGTAATCCTGTCGAAAAGAACATACCTTTCGCACTACCACTATGGGATCTCCTCCGGAGATATCAGTGGATTGAATTCGCATTCACGCTTCTCGATCACATCCTTGCCTTGTTTGGAGGTCTACGAAGGTGGCCATGCCGCCAGGTAGCCCTGAAAAGGTGTACCGCATGGCTGCTCGAGCATCAGGAGGAGTCTGGCGATTGGGCTGGCTTTTTTCCTCCGATCCATGGCAGTATATGGGCACTGCTCCTTGATGGCTTTTCGTTCCAATCCGAAACCATCCGTCTAGGCATGGAAGCATTGGAACGTCTTGTCATTGTCGACCCGAAAGGAAGATGGGTGCAATCCACAGTATCTCCCTGCTGGGACACAGCTCTCATGGCGAATGCGTTATGCGATGCCGGCATGAGCGGCGATACTCGTCTGGCAAAAGCAACGCAATGGCTTCGAGACCGACAGCTGATGGTCTCTCACGGTGACTGGCGCAACTATGCAAACACCCAACAGGCTGGAGGGTGGAGCTTCCAGTATTTCAATTCATTCTATCCGGACGTCGACGACACAGCAGTCGTAATAATGACACTCATCAAGGAAGATCCAAGTTGCACCAATTCTGACTGCGTGATGAATGGCGTTGAATGGATGCTTGGAATGCAGAGCCGAGACGGCGGGTGGGGGGCTTTCGATGTGAACAACGACGCACGCTGGTTGCACAAGATTCCCTTCAGTGACATGGACAGTCTCGTAGACCCAAGCACGTCAGATGTCACTGGACGGATCTTGGAATGCCTTGGTCTGTTGCTATCGCAGAGAAAAAGCCCTCTGTTACCGCATTGGAAACATCGTCTTCAAGCGTCCTCCGCGAGAGCAATCGCTTTCCTCGCCAAAGAACAAGAGCCCTCAGGCGCGTGGTGGGGCAGATGGGGCAACAATTACCACTATGGTACCGCAAACGTTCTCCGAGGCCTAGCTTGCTTCGCACAAACTGACCCAAATGCACAAGTTATGTGCATGCGTACGCTTTCCTGGATCGACGGGACTCAGAACGCTGATGGTGGTTGGGGGGAAACACTAGCATCATACGTCGATAACTCACTGGCAGGTCTCGGTAGCAGCACTGCCGCACACACTGCCTGGGCGCTCGAGTCCCTGCTACGCTTCCGTCTACCATCTGATCAAGCTATCGAGCGAGGTGTGCGATGGCTCGTTGACAATCAGCAACCGAATGTAGACGGCTACTACTACGGAACGAAATGGCAGACAGGCACAGGTCAAGGAGCATCCTGGCGCTTCGACCATGCCTATGTCGGTACAGGATTTCCAAGTGTTCTTTATCTGGGCTATCCTTATTACCATCATCTGTTTCCGATCCAAGCCCTGAGTCGTTACATCGACAAGGCAAGTCGTCAAGGGCTCGAGACATTGAGGGTACCATCCTCATCTGCCGCCTTCATTGACCATCCAAACGTGCTCTTGATGGCCATGGGCAGTCATGGTGACATTCAAGTATTCTTGAACGTTGCCAAAAGACTTTCGGGTTGTCGTGTCCGGATTGCCACACACCCCGCTCACCAAGCCAAGGTCGAGGGACACGGCTTTGAGTTTTACGACGTGGGTGGCAGCCCCGAAGTCTTCTCTGCTGCCCTTGCCAATGAACATGGGATCCTCCGGTCGATCGTTGACGGCAGCTTTCGTGAGCTTCAACACTCGCTGTGTTCGATTTACAAGAGCTTCTGGGTTGCTGCACTCGACGATGTGCAATCCTATTCACCATTGAAGCCAGAACCATCGTCCCGGCCATTTATCGCTGATGTCGTTGTGTCGGGTCCGTCTACCTCGGTTCATATACATGCTGCGGAGAGAGCTCAGGCACCACTGGTTATCATTTCGACACAGCCAGCCATCATCACCGGCGACTTTCAATCTCCACTTACGATGTCACGGGCTCAATCCAACCCAGGTAGGCTCTGGAACAAGTTATCTTTCTACATACTAGCCTTTTTGTAAGTCCTATCCACACTCATACAGTATCGCGACGATGTCTAACCAAGATAGTGACTGGCTCTCATTTGGTCCATCCTTCACCCGCATGCGAGCTAATAGTTACCAACTCCGGAGCCTAGATCTGGTCTGGGCACTGTTTGAACTTGTCAAGGTCAATGTACCACATGTATGTCTGTGGTCTGCTTCTCTCGTCCCAAAGCCTGAGGACTGGAGCAACAATGTTGTCATTGCAGGCTACAGCCCTATGAGTGATGACGCAGACTATGTCGCCTCGAACACACTGCAGGCTTTCTTGGAGACTCGACAACCCGTGGTTGCCATCAGCTTCGGCTCAGCCACCATCGAGGATCCAATGAATCTCATCAAAACTATTGCAGCTGCTCTATCAAAGGTCGGAGCAAGCTCTGTCCTTTGCCGGAGCTGGGACTCTTCTTTCGAGGCAGAAGCAGACCTTCCTTCAAATGTGTTTCTCGTCGATTCAATTCCACATGGCTGGCTTCTACACCACGTGAAAGGATTCATTCATCACGGTGGAGCTGGACATACAGCTGCTGGTGCAAAAGCAGGCGTACCTCAGCTGGTCGTGCCGCAATTTCTCGATCAATTCTTCTGGGCAGCCAAAGTATCTGAAATGGGTCTAGGACCAACGCCCCTACCTCTTCGTGAACTTTCTCTGGATGAGCTAGCACCACGCATGGAGGACCTACTTTCGTCAAAGTACACTAAAGCTTGCACAAACATGGCTTTACAGCTTCGGGGTGATGTCGATGGCGCAGATGTTGCCGGCGATGAGATCTTGCGCCAAGTGGAAGTCATCACGACTTGCCGCATATTCCCAGAGCTCAGTGCTCACTGGTACTATGCTGAATCGAACCTGAGCCTCTCTGGAGTTGCTGCTGCAAGCCTGGTATCGTCCAAAGAGATCCAATGGCAAGATCTGGAACTACGACCAGCGAAGGACTGGGAGCAACAATGGCGCACCGTACAATCATCTTCTCAACTAGTCAAGATTTGGCGCGCGGTCGTGCAACTACCCTATTTGTTTACAACTATGATTCCTGCGTTCTTTGGCTGGCTCAAAGGTACGCATGGCTACTTGGACGGTGACAGGTACGTCATCAAGATGGAAGATCCTTTGCGGCAAGCCAGGTTGGAGCAAGCTGAGTTTGATCTGCACCTCATACACCAAGCATACGATTCTGGAAATTCTACCAGTCTCGATGCCAAAATCATCGAAAATTGGAAGGCAAGGAAGGCTATCGCGATTCATGAAGTCTTTGACGATGATACTAGCGCGAGTGAAAGTTCAAGATCGTCTTTGGATGGTGGACATGCTGATAGTGTGCTTGATATCGAAGAGAAGTAG

Note S3. Isolation and purification of metabolites

Purification process for **5** and **6**

Mycelia and culture medium from 5 L culture of *AO-efuA_(TC)_fsoA_(GT)_-fsoD/E/F* were extracted with ethanol and EtOAc, respectively. The crude extract was fractionated by MPLC using the ODS column with stepwise elution of MeOH and H_2_O. The fraction containing **5** and **6** was further purified by semi-preparative HPLC using the YMC Pack ODS-A column with isocratic elution of 70% CH_3_CN–H_2_O containing 0.1% formic acid (3 mL/min) to yield **5** (1.0 mg) and **6** (15 mg).

Purification process for **7**

Mycelia from 5 L culture of *AO-efuA_(TC)_-fsoD* were extracted with ethanol. The crude extract was fractionated by silica gel column chromatography with stepwise elution of cyclohexane and ethyl acetate. The fraction containing **7** was further purified by semi-preparative HPLC using the YMC Pack ODS-A column with isocratic elution of 100% MeOH containing 0.1% formic acid (3 mL/min) to yield **7** (15 mg).

Purification process for **8**

Mycelia and culture medium from 10 L culture of *AO-efuA_(TC)_fsoA_(GT)_-fsoD/F* were extracted with ethanol and EtOAc, respectively. The crude extract was fractionated by MPLC using the ODS column with stepwise elution of MeOH and H_2_O. The fraction containing **8** was further purified by semi-preparative HPLC using the YMC Pack ODS-A column with isocratic elution of 40% CH_3_CN–H_2_O containing 0.1% formic acid (3 mL/min) to yield **8** (10 mg).

Purification process for **9**

Mycelia and culture medium from 5 L culture of *AO-efuA_(TC)_-fsoD/F* were extracted with ethanol and EtOAc, respectively. The crude extract was fractionated by MPLC using the ODS column with stepwise elution of MeOH and H_2_O. The fraction containing **9** was further purified by semi-preparative HPLC using the YMC Pack ODS-A column with isocratic elution of 100% MeOH containing 0.1% formic acid (3 mL/min) to yield **9** (1.4 mg).

Supplementary Tables

Table S1. Primers used in the study

| **Primer** | **Sequence (5′ to 3′)** | **Usage** |
| --- | --- | --- |
| Inf-pTA-efmAtc-F | TCGAGCTCGGTACCCACACACAATGGACATGGCGC | Cloning of *efmA_(TC)_* and *efmA* for construction of pTAex3-*efmA_(TC)_* and pTAex3-*efmA* |
| Inf-pTA-efmAtc-R | CTACTACAGATCCCCTCAAACGTGCCGAGTCATGAG |
| Inf-pTA-efmAgt-R | CTACTACAGATCCCCCTACTTCTCTTCGATATCAAG |
| Inf-pTA-efuAtc-F | TCGAGCTCGGTACCCATGCCGTCTTACCACAACAC | Cloning of *efuA_(TC)_* and *efuA_(GT)_* for construction of pTAex3-*efuA* |
| Inf-uAtc-uAgt-R | CCACGACTACCCATGGCCATCAAGAGTACGTTCGGACGGTC |
| Inf-uAtc-uAgt-F | CCGTCCGAACGTACTCTTGATGGCCATGGGTAGTCGTGG |
| Inf-pTA-uAgt-R | CTACTACAGATCCCCCTACTTCTCTTCGATATCAAGCACAC |
| Inf-pTA-fosAtc-F | TCGAGCTCGGTACCCATGGACATGGCGCCAGACGAG | Cloning of *fsoA_(TC)_* and *efuA_(GT)_* for construction of pTAex3-*fsoA_(TC)_efuA_(GT)_* |
| Inf-oAtc-uAgt-R | CCACGACTACCCATGGCCATGAGGAGCGGCAGATCACGGT |
| Inf-oAtc-uAgt-F | ACCGTGATCTGCCGCTCCTCATGGCCATGGGTAGTCGTGG |
| Inf-pTA-uAgt-R | CTACTACAGATCCCCCTACTTCTCTTCGATATCAAGCACAC |
| FsoA-TC-D412/143A-F | ACACCTGGTACCCAGATGTGGCCGCCACGGCGGTTGTGGTCATG | Mutation of *fsoA* for construction of pTAex3-*fsoA_(dTC-GT)_* |
| FsoA-TC-D412/143A-R | TGGGTACCAGGTGTTGTTGT |
| Inf-pTA-GUS-F | TCGAGCTCGGTACCCATGGTAGATCTGAGGGTAAATTT | Cloning of *GUS* for construction of pTAex3-*GUS* |
| Inf-pTA-GUS-R | CTACTACAGATCCCCTTGTTTGCCTCCCTGCTG |
| Inf-pTA-efuI-F | TCGAGCTCGGTACCCATGACAGCAGCAGTCCAGTC | Cloning of *efuI* for construction of pTAex3-*efuI* |
| Inf-pTA-efuI-R | CTACTACAGATCCCCTTACTCCCTCTCTTGATCCTCCC |
| Inf-NotI-U6pro-F | CTGAGGGTTTAATTAATGGTTCACTTCTCTTTAG | Cloning of sgRNA scaffold for construction of pSC-HS601-gRNA, pSC-HS801-gRNA, and pSC-*ku80*-gRNA |
| pSC-gRNA-mut-R | CACTTGTTCTTCTTTACAATGATTTA |
| U6ter-HS601-F | GAAGAACAAGTGTCGGAGTATAGCGGTCCACCGTTTTAGAGCTAGAAATAGC |
| U6ter-HS801-F | CATTGTAAAGAAGAACAAGTGCTTTGAAGTGAACTACTGCTGTTTTAGAGCTAGAAATAGC |
| U6ter-ku80-F | TTGTAAAGAAGAACAAGTGGGTTCTCACAGTGGTCAATCGTTTTAGAGCTAGAAATAGC |
| Inf-NotI-U6ter-R | CTACAGGGCGCGTGCGGCCCAGCAGCTCTATATCAC |
| HS401-up-F | cgactcactatagggcccGGAAATAGCCACCATTCCC | Cloning of homologous arms for construction of pESCa-HS401 |
| HS401-up-R | GAGTATGAATAAGTCGAATGGcccgggGTGTTGTTGCTTATCGTCCG |
| HS401-down-F | CGGACGATAAGCAACAACACcccgggCCATTCGACTTATTCATACTCAGC |
| HS401-down-R | cttctgttccatgtcgacgcccGCCTTTGTCTAGGGATATTTCTAC |
| HS601-up-F | ctcactatagggcccGTCCTCCTGGGCAGTGTAG | Cloning of homologous arms for construction of pESCa-HS601 |
| HS601-up-R | TGGACTCCTTTCCCcccgggTTGTTTAATGGGGAGGTCGAATG |
| HS601-down-F | CTCCCCATTAAACAAcccgggGGGAAAGGAGTCCACATTC |
| HS601-down-R | tccatgtcgacgcccACAACGAGTGGCAGGAG |
| HS801-up-F | ctcactatagggcccAAGAAAAGGCGGTGAACTAAC | Cloning of homologous arms for construction of pESCa-HS801 |
| HS801-up-R | ctcactatagggcccAAGAAAAGGCGGTGAACTAAC |
| HS801-down-F | TTACCCATTGCTTGGcccgggTCGAAGGGTATCATCAATGTGTAG |
| HS801-down-R | tccatgtcgacgccccTTTGGGAAAAACCTCGTGGAT |
| Ku80-up-F | ctcactatagggccccCTTCATTGGAAAGGACGAGG | Cloning of homologous arms for construction of pESCa*-ku80* |
| Ku80-up-R | GAATGTATCGAGAATcccgggTAGAAAAGAACAAAGCTGCG |
| Ku80-down-F | CTTTGTTCTTTTCTAcccgggATTCTCGATACATTCGCAGG |
| Ku80-down-R | tccatgtcgacgcccTCAATCTGGAACCACCTGC |
| Inf-HS401-F | CGATAAGCAACAACACCCCGCAAGAGCTCAACTCCTATTC | Cloning of target genes under the *amyB* promoter for construction of pESCa-HS401-based plasmids |
| Inf-HS401-R | GTATGAATAAGTCGAATGGCCCGGAAACATCATGGTGGGG |
| Inf-HS601-F | CCTCCCCATTAAACAACCCCCAATCTTCAAGAGCAGAATG | Cloning of target genes under the *amyB* promoter for construction of pESCa-HS601-based plasmids |
| Inf-HS601-R | GAATGTGGACTCCTTTCCCCCCGTAAGATACATGAGCTTCGGTG |
| Inf-HS601-4-F | CCTCCCCATTAAACAAcccCCAAGCTCTAATACGACTCAC |
| Inf-HS601-4-R | GAATGTGGACTCCTTTCCCcccCGAACGAGGAGCCATATTTTG |
| Inf-HS601-4-F1 | gattgaaggcgttgcgttcCAATACGCAAACCGCCTC |
| Inf-HS601-4-R1 | GAGGCGGTTTGCGTATTGgaacgcaacgccttcaatc |
| Inf-HS801-F | TTACCCATTGCTTGGcccCCAATCTTCAAGAGCAGAATG | Cloning of target genes under the *amyB* promoter for construction of pESCa-HS801-based plasmids |
| Inf-HS801-R | CATTGATGATACCCTTCGAcccTGTAAGATACATGAGCTTCGGTG |
| Inf-pET28b-efuI-F | GTACTTCCAGTCACATATGACAGCAGCAGTCCAGTC | Cloning of intron-free *efuI* for construction of the pET28b-*efuI* plasmid |
| Inf-pET28b-efuI-R | AGTGCGGCCGCAAGCTTTACTCCCTCTCTTGATCCTCCC |
| overlap-efuI-F1 | GATTGACCTAATCAAAGAAACTACGGGCGCTAGCTTTGTG |
| overlap-efuI-R1 | CAGCACAAAGCTAGCGCCCGTAGTTTCTTTGATTAGGTCAATCAGC |
| overlap-efuI-F2 | CCAGCAATGTCTGTACACATTGACCAAACCCCAGAGGGCGC |
| overlap-efuI-R2 | CAGCGCCCTCTGGGGTTTGGTCAATGTGTACAGACATTGCTGG |

Table S2. Plasmids used in the study

| **Plasmid** | **Characteristic** | **Source** |
| --- | --- | --- |
| pTAex3 | Plasmid containing the *amyB* promoter and terminator, as well as *argB* marker gene cassette, for gene expression in *A. oryzae* NSAR1. | Fujii T., *et al.*^[1]^ |
| pUSA | Plasmid containing the *amyB* promoter and terminator, as well as *sC* marker gene cassette, for gene expression in *A. oryzae* NSAR1. | Yamada O., *et al.*^[2]^ |
| pAdeA | Plasmid containing *adeA* marker gene cassette for gene expression in *A. oryzae* NSAR1. | Jin F. J., *et al.*^[3]^ |
| pTAex3-*efuA* | pTAex3 containing *efuA* under the *amyB* promoter. | Cao Z. Q., *et, al.*^[4]^ |
| pTAex3-*efuA_(TC)_* | pTAex3 containing *efuA_(TC)_* under the *amyB* promoter. |
| pTAex3-*efuA_(TC)_fsoA_(GT)_* | pTAex3 containing *efuA_(TC)_fsoA_(GT)_* under the *amyB* promoter. |
| pTAex3-*fsoD* | pTAex3 containing *fsoD* under the *amyB* promoter. |
| pTAex3-*fsoF* | pTAex3 containing *fsoF* under the *amyB* promoter. |
| pUSA-*fsoE* | pUSA containing *fsoE* under the *amyB* promoter. |
| pAdeA-*fsoD/F* | pAdeA containing *fsoD* and *fsoF*, the expression of which is individually regulated by the *amyB* promoter. |
| pTAex3-*efmA* | pTAex3 containing *efmA* under the *amyB* promoter. | This work |
| pTAex3-*efmA_(TC)_* | pTAex3 containing *efmA_(TC)_* under the *amyB* promoter. |
| pTAex3-*fsoA_(TC)_efuA_(GT)_* | pTAex3 containing *fsoA_(TC)_efuA_(GT)_* under the *amyB* promoter. |
| pTAex3-*fsoA_(dTC-GT)_* | pTAex3 containing *fsoA_(dTC-GT)_* under the *amyB* promoter. |
| pTAex3-*GUS* | pTAex3 containing *GUS* under the *amyB* promoter. |
| pTAex3-*efuI* | pTAex3 containing *efuI* under the *amyB* promoter. |
| pSC-134 | Plasmid containing *AMA1*, *cas9* cassette and *pyrG* marker gene cassette. | Yuan Y., *et al.*^[5]^ |
| pSC-HS401-gRNA | Plasmid containing *AMA1*, *cas9* cassette, *pyrG* marker gene cassette and *HS401-sgRNA* cassette for cutting *HS401*. |
| pSC-HS601-gRNA | Plasmid containing *AMA1*, *cas9* cassette, *pyrG* marker gene cassette and HS601-sgRNA cassette for cutting *HS601.* | This work |
| pSC-HS801-gRNA | Plasmid containing *AMA1*, *cas9* cassette, *pyrG* marker gene cassette and *HS801-sgRNA* cassette for cutting *HS801*. |
| pSC-*ku80*-gRNA | Plasmid containing *AMA1*, *cas9* cassette, *pyrG* marker gene cassette and *ku80-sgRNA* cassette for cutting *ku80*. |
| pESC-Ura | Plasmid containing *ura3* marker gene cassette for gene expression in *S. cerevisiae*. | Agilent Technologies, Inc. |
| pESCa-HS401 | Plasmid providing the donor fragment that is site-specifically integrated into HS401 locus through homologous recombination. | This work |
| pESCa-HS601 | Plasmid providing the donor fragment that is site-specifically integrated into HS601 locus through homologous recombination. |
| pESCa-HS801 | Plasmid providing the donor fragment that is site-specifically integrated into HS801 locus through homologous recombination. |
| pESCa-*ku80* | Plasmid providing the donor fragment that is site-specifically integrated into *ku80* locus through homologous recombination. |
| pESCa-HS401-*efuA* | Plasmid providing the donor fragment containing the *efuA* expression cassette that is site-specifically integrated into *HS401* locus through homologous recombination. |
| pESCa-HS401-*efuA_(TC)_* | Plasmid providing the donor fragment containing the *efuA_(TC)_* expression cassette that is site-specifically integrated into *HS401* locus through homologous recombination. |
| pESCa-HS401-*efuA_(TC)_fsoA_(GT)_* | Plasmid providing the donor fragment containing the *efuA_(TC)_fsoA_(GT)_* expression cassette that is site-specifically integrated into *HS401* locus through homologous recombination. |
| pESCa-HS401-*efmA* | Plasmid providing the donor fragment containing the *efmA* expression cassette that is site-specifically integrated into *HS401* locus through homologous recombination. |
| pESCa-HS401-*efmA_(TC)_* | Plasmid providing the donor fragment containing the *efmA_(TC)_* expression cassette that is site-specifically integrated into *HS401* locus through homologous recombination. |
| pESCa-HS401-*fsoA_(TC)_* | Plasmid providing the donor fragment containing the *fsoA_(TC)_* expression cassette that is site-specifically integrated into *HS401* locus through homologous recombination. |
| pESCa-HS401-*fsoA* | Plasmid providing the donor fragment containing the *fsoA* expression cassette that is site-specifically integrated into *HS401* locus through homologous recombination. |
| pESCa-HS401-*fsoA_(TC)_efuA_(GT)_* | Plasmid providing the donor fragment containing the *fsoA_(TC)_efuA_(GT)_* expression cassette that is site-specifically integrated into *HS401* locus through homologous recombination. |
| pESCa-HS601-*fsoD* | Plasmid providing the donor fragment containing the *fsoD* expression cassettes that is site-specifically integrated into *HS601* locus through homologous recombination. |
| pESCa-HS601-*fsoD/F* | Plasmid providing the donor fragment containing the *fsoD* and *fsoF* expression cassettes that is site-specifically integrated into *HS601* locus through homologous recombination. |
| pESCa-HS601-*fsoD/E/F* | Plasmid providing the donor fragment containing the *fsoD*, *fsoE*, and *fsoF* expression cassettes that is site-specifically integrated into *HS601* locus through homologous recombination. |
| pESCa-HS801-*fsoA_(dTC-GT)_* | Plasmid providing the donor fragment containing the *fsoA_(dTC-GT)_* expression cassette that is site-specifically integrated into *HS801* locus through homologous recombination. |
| pESCa-HS801-*fsoA_(GT)_* | Plasmid providing the donor fragment containing the *fsoA_(GT)_* expression cassette that is site-specifically integrated into *HS801* locus through homologous recombination. |
| pESCa-HS801-*GUS* | Plasmid providing the donor fragment containing the *GUS* expression cassette that is site-specifically integrated into *HS801* locus through homologous recombination. |
| pESCa-HS801-*efuI* | Plasmid providing the donor fragment containing the *efuI* expression cassette that is site-specifically integrated into *HS801* locus through homologous recombination. |
| pET28b | Plasmid containing the T7 promoter and terminator, as well as maltose-binding protein (MBP) and 6×His tag, for protein expression in *E.coli*. | Novagen, Inc. |
| pET28b-*efuI* | Plasmid containing *efuI* coding sequence under T7 promoter, for protein expression in *E.coli*. | This work |

Table S3. Strains used in the study

| **Strain** | **Characteristic** | **Source** |
| --- | --- | --- |
| *A. oryzae* S184 | The *∆pyrG* mutant strain derived from *A. oryzae* NSAR1 | Yuan Y., *et al.*^[5]^ |
| *A. oryzae* J001 | The *∆ku80* mutant strain derived from *A. oryzae* S184 | This work |
| *A. oryzae* S184-*GUS* | *A. oryzae* S184 transformant harboring *GUS* reporter gene |
| *A. oryzae* J001-*GUS* | *A. oryzae* J001 transformant harboring *GUS* reporter gene |
| *A. oryzae* J002 | The engineered *A. oryzae* J001 with the optimized MVA pathway |
| *AO-efuA_(TC)_* | *A. oryzae* J002 transformant harboring *efuA_(TC)_* |
| *AO-efuA* | *A. oryzae* J002 transformant harboring *efuA* |
| *AO-efmA_(TC)_* | *A. oryzae* J002 transformant harboring *efmA_(TC)_* |
| *AO-efmA* | *A. oryzae* J002 transformant harboring *efmA* |
| *AO-efuA_(TC)_fsoA_(GT)_* | *A. oryzae* J002 transformant harboring *efuA_(TC)_fsoA_(GT)_* |
| *AO-fsoA* | *A. oryzae* J002 transformant harboring *fsoA* |
| *AO-fsoA_(TC)_efuA_(GT)_* | *A. oryzae* J002 transformant harboring *fsoA_(TC)_efuA_(GT)_* |
| *AO-efuA_(TC)_fsoA_(GT)_-fsoD/E/F* | *A. oryzae* J002 transformant harboring *efuA_(TC)_fsoA_(GT)_*, *fsoD*, *fsoE* and *fsoF* |
| *AO-efuA-fsoD/E/F* | *A. oryzae* J002 transformant harboring *efuA*, *fsoD*, *fsoE* and *fsoF* |
| *AO-efuA_(TC)_-fsoD* | *A. oryzae* J002 transformant harboring *efuA_(TC)_* and *fsoD* |
| *AO-efuA_(TC)_fsoA_(GT)_-fsoD* | *A. oryzae* J002 transformant harboring *efuA_(TC)_fsoA_(GT)_ and fsoD* |
| *AO-efuA_(TC)_-fsoD/F* | *A. oryzae* J002 transformant harboring *efuA_(TC)_*, *fsoD* and *fsoF* |
| *AO-efuA_(TC)_fsoA_(GT)_-fsoD/F* | *A. oryzae* J002 transformant harboring *efuA_(TC)_fsoA_(GT)_*, *fsoD and fsoF* |
| *AO-efuA_(TC)_-fsoD/F-fsoA_(dTC-GT)_* | *A. oryzae* J002 transformant harboring *efuA_(TC)_*, *fsoD*, *fsoF and fsoA_(dTC-GT)_* |
| *AO-efuA_(TC)_-fsoD/F-fsoA_(GT)_* | *A. oryzae* J002 transformant harboring *efuA_(TC)_, fsoD, fsoF and fsoA_(GT)_* |
| *AO-fsoA_(dTC-GT)_* | *A. oryzae* J001 transformant harboring *fsoA_(dTC-GT)_* |
| *AO-fsoA_(TC)_-fsoD* | *A. oryzae* J002 transformant harboring *fsoA_(TC)_ and fsoD* |
| *AO-fsoA_(TC)_-fsoD/F* | *A. oryzae* J002 transformant harboring *fsoA_(TC)_, fsoD and fsoF* |
| *AO-fsoA-fsoD* | *A. oryzae* J002 transformant harboring *fsoA and fsoD* |
| *AO-fsoA-fsoD/F* | *A. oryzae* J002 transformant harboring *fsoA, fsoD and fsoF* |
| *AO-fsoE* | *A. oryzae* J001 transformant harboring *fsoE* |
| *AO-efuA_(TC)_fsoA_(GT)_-fsoD/E/F-efuI* | *A. oryzae* J002 transformant harboring *efuA_(TC)_fsoA_(GT)_*, *fsoD*, *fsoE,* *fsoF* and *efuI* |

Table S4. NMR assignments for 5 (^1^H for 600 MHz and ^13^C for 150 MHz in pyridine-*d*_5_)

| No. | *δ*_C_, type | *δ*_H_ (*J* in Hz)*^a^* | ^1^H-^1^H COSY | HMBC | ROESY |
| --- | --- | --- | --- | --- | --- |
| 1 | 45.6, CH_2_ | a: 2.36 | 1b, 2 | 2, 3, 5, 10, 25 | 11, 25 |
|  |  | b: 1.58 | 1a, 2 | 2, 3, 5, 9, 10, 25 | 3, 11 |
| 2 | 70.0, CH | 5.61, ddd (12.6, 10.2, 4.2) | 1a, 1b, 3 | 3, 2-COCH_3_ | 25 |
| 3 | 88.6, CH | 3.63, d (10.0) | 2 | 1, 2, 4, 23, 24, 1′ | 1b, 5, 23, 1′ |
| 4 | 41.4, C |  |  |  |  |
| 5 | 44.2, CH | 1.51 | 6a, 6b | 3, 4, 6, 9, 10, 24, 25 | 3, 8 |
| 6 | 19.1, CH_2_ | a: 1.70 | 5, 6b, 7a, 7b | 5, 7, 10 |  |
|  |  | b: 1.57 | 5, 6a, 7a, 7b | 5, 7, 8, 10 |  |
| 7 | 18.1, CH_2_ | a: 1.60 | 6a, 6b, 7b, 8 | 5, 8, 9 |  |
|  |  | b: 1.26 | 6a, 6b, 7a, 8 |  |  |
| 8 | 39.2, CH | 2.11 | 7a, 7b. 11 |  | 5, 27 |
| 9 | 150.3, C |  |  |  |  |
| 10 | 38.9, C |  |  |  |  |
| 11 | 116.7, CH | 5.39 | 8, 12a, 12b | 8, 10, 13 | 1a, 1b, 25 |
| 12 | 35.4, CH_2_ | a: 2.83, dd (17.4, 5.4) | 11, 12b | 9, 11, 13, 14, 27 | 27 |
|  |  | b: 1.82, br d (16.8) | 11, 12a | 9, 13 | 18, 26 |
| 13 | 36.6, C |  |  |  |  |
| 14 | 37.5, C |  |  |  |  |
| 15 | 29.0, CH_2_ | a: 1.40 | 15b, 16a, 16b |  |  |
|  |  | b: 1.30 | 15a, 16a, 16b |  |  |
| 16 | 35.9, CH_2_ | a: 1.72 | 15a, 15b, 16b | 14, 28 |  |
|  |  | b: 1.61 | 15a, 15b, 16a | 28 | 18 |
| 17 | 43.0, C |  |  |  |  |
| 18 | 64.3, CH | 2.26, s |  | 12, 13, 17, 19, 21, 27, 28 | 12b, 16b, 21, 26 |
| 19 | 214.4, C |  |  |  |  |
| 20 | 42.5, CH_2_ | a: 2.36 | 20b, 21 | 17, 18, 19, 21 | 29 |
|  |  | b: 1.75 | 20a, 21 | 19, 21, 22 |  |
| 21 | 54.9, CH | 1.34 | 20a, 20b, 22 | 16, 28 | 18 |
| 22 | 30.3, CH | 1.47 | 21, 29, 30 |  | 28 |
| 23 | 27.8, CH_3_ | 1.37, s |  | 3, 4, 5, 24 | 3, 1′ |
| 24 | 17.4, CH_3_ | 1.21, s |  | 3, 4, 5, 23 |  |
| 25 | 26.2, CH_3_ | 1.18, s |  | 1, 5, 9, 10 | 1a, 2, 11 |
| 26 | 15.9, CH_3_ | 0.78, s |  | 8, 13, 14, 15 | 12b, 18 |
| 27 | 17.3, CH_3_ | 1.19. s |  | 12, 13, 14, 18 | 8, 12a, 28 |
| 28 | 15.8, CH_3_ | 0.89, s |  | 16, 17, 18, 21 | 22, 27 |
| 29 | 22.9, CH_3_ | 0.81, d (6.6) | 22 | 21, 22, 30 | 20a |
| 30 | 22.3, CH_3_ | 0.93, d (6.6) | 22 | 21, 22, 29 |  |
| 1′ | 106.2, CH | 4.98 | 2′ | 3, 3′, 5′ | 3, 23, 3′, 5′ |
| 2′ | 75.8, CH | 4.02 | 1′, 3′ | 1′, 3′ |  |
| 3′ | 78.7, CH | 4.24, t (9.0) | 2′, 4′ | 2′, 4′ | 1′ |
| 4′ | 72.1, CH | 4.18, t (9.0) | 3′, 5′ | 3′, 5′, 6′ | 6′b |
| 5′ | 78.1, CH | 4.01 | 4′, 6′a, 6′b | 1′, 3′, 4′ | 1′, 2-COCH_3_ |
| 6′ | 63.2, CH_2_ | a: 4.60, dd (11.4, 2.4) | 5′, 6′b |  | 2-COCH_3_ |
|  |  | b: 4.40, dd (11.4, 5.4) | 5′, 6′a | 4′, 5′ | 4′, 2-COCH_3_ |
| 2-COCH_3_ | 171.1, C |  |  |  |  |
| 2-COCH_3_ | 21.8, CH_3_ | 2.46, s |  | 2-COCH_3_ | 5′, 6′a, 6′b |

*^a^* The indiscernible signals from overlap or the complex multiplicity are reported without designating multiplicity.

Table S5. NMR assignments for 6 (^1^H for 600 MHz and ^13^C for 150 MHz in pyridine-*d_5_*)

| No. | *δ*_C_, type | *δ*_H_ (*J* in Hz)*^a^* | ^1^H-^1^H COSY | HMBC | ROESY |
| --- | --- | --- | --- | --- | --- |
| 1 | 45.5, CH_2_ | a: 2.37, dd (12.6, 4.8) | 1b, 2 | 2, 3, 5, 10, 25 | 11, 25 |
|  |  | b: 1.58 | 1a, 2 | 2, 3, 5, 9, 10, 25 | 3, 11 |
| 2 | 70.0, CH | 5.60, ddd (12.0, 9.6, 4.2) | 1a, 1b, 3 | 1, 3, 2-COCH_3_ | 24, 25 |
| 3 | 88.6, CH | 3.62, d (10.2) | 2 | 1, 2, 4, 5, 23, 24, 1′ | 1b, 5, 23, 1′, 2-COCH_3_ |
| 4 | 41.4, C |  |  |  |  |
| 5 | 44.2, CH | 1.52 | 6a, 6b | 3, 9, 25 | 3, 8 |
| 6 | 19.1, CH_2_ | a: 1.66 | 5, 6b, 7a, 7b | 5, 10 |  |
|  |  | b: 1.53 | 5, 6a, 7a, 7b |  |  |
| 7 | 17.9, CH_2_ | a: 1.69 | 6a, 6b, 7b, 8 | 5, 9 |  |
|  |  | b: 1.29 | 6a, 6b, 7a, 8 | 14 |  |
| 8 | 40.4, CH | 2.22, br d (13.8) | 7a, 7b, 11 | 9, 11 | 5 |
| 9 | 150.5, C |  |  |  |  |
| 10 | 38.9, C |  |  |  |  |
| 11 | 116.7, CH | 5.37 | 8, 12a, 12b | 8, 9, 12 | 1a, 1b, 25 |
| 12 | 38.2, CH_2_ | a: 2.27, br d (17.4) | 11, 12b | 9, 11, 13, 18, 27 | 18, 26 |
|  |  | b: 1.99, dd (17.4, 5.1) | 11, 12a | 9, 11, 13, 14, 27 | 27 |
| 13 | 38.1, C |  |  |  |  |
| 14 | 37.5, C |  |  |  |  |
| 15 | 28.2, CH_2_ | a: 1.52 | 15b, 16a, 16b |  |  |
|  |  | b: 1.39 | 15a, 16a, 16b | 13, 16 | 26 |
| 16 | 29.6, CH_2_ | a: 1.69 | 15a, 15b, 16b | 15, 17, 21, 28 | 22 |
|  |  | b: 1.29 | 15a, 15b, 16a | 14 |  |
| 17 | 40.9, C |  |  |  |  |
| 18 | 52.9, CH | 3.19, s |  | 12, 13, 16, 17, 19, 21, 27, 28 | 12a, 21, 22, 26 |
| 19 | 176.7, C |  |  |  |  |
| 20 | 8.4, CH_3_ | 0.87, d (7.2) | 21 | 17, 21, 22 | 28 |
| 21 | 48.2, CH | 1.60 | 20, 22 | 16, 17, 18, 20, 22, 29, 30 | 18, 29 |
| 22 | 26.8, CH | 2.51 | 21, 29, 30 | 17, 20, 21, 29, 30 | 16a, 18 |
| 23 | 27.8, CH_3_ | 1.38, s |  | 3, 4, 5, 24 | 3, 1′, 2′ |
| 24 | 17.4, CH_3_ | 1.21, s |  | 3, 4, 5, 23 | 2, 2′ |
| 25 | 26.2, CH_3_ | 1.18, s |  | 1, 5, 9, 10 | 1a, 2, 11 |
| 26 | 15.6, CH_3_ | 0.89, s |  | 8, 13, 14, 15 | 12a, 15b, 18 |
| 27 | 17.3, CH_3_ | 1.55. s |  | 12, 13, 14, 18 | 12b |
| 28 | 20.9, CH_3_ | 1.53, s |  | 16, 17, 18, 21 | 20 |
| 29 | 25.1, CH_3_ | 1.02, d (6.6) | 22 | 21, 22, 30 | 21 |
| 30 | 18.6, CH_3_ | 0.93, d (6.6) | 22 | 21, 22, 29 |  |
| 1′ | 106.2, CH | 4.98, d (7.8) | 2′ | 3, 2′, 3′, 5′ | 3, 23, 3′, 5′ |
| 2′ | 75.8, CH | 4.00 | 1′, 3′ | 1′, 3′ | 23, 24 |
| 3′ | 78.7, CH | 4.24, t (8.4) | 2′, 4′ | 1′, 2′, 4′ | 1′ |
| 4′ | 72.1, CH | 4.17, t (9.2) | 3′, 5′ | 3′, 5′, 6′ |  |
| 5′ | 78.1, CH | 4.00 | 4′, 6′a, 6′b | 1′, 3′, 6′ | 1′, 2-COCH_3_ |
| 6′ | 63.2, CH_2_ | a: 4.59, dd (11.4, 2.4) | 5′, 6′b | 4′, 5′ | 2-COCH_3_ |
|  |  | b: 4.39, dd (11.4, 5.4) | 5′, 6′a | 4′, 5′ | 2-COCH_3_ |
| 2-COCH_3_ | 171.0, C |  |  |  |  |
| 2-COCH_3_ | 21.8, CH_3_ | 2.43, s |  | 2-COCH_3_ | 3, 5′, 6′a, 6′b |

*^a^* The indiscernible signals from overlap or the complex multiplicity are reported without designating multiplicity.

**Table S6.** NMR assignments for **7** (^1^H for 600 MHz and ^13^C for 150 MHz in CDCl_3_)

| No. | *δ*_C_, type | *δ*_H_ (*J* in Hz)*^a^* | ^1^H-^1^H COSY | HMBC | ROESY |
| --- | --- | --- | --- | --- | --- |
| 1 | 47.2, CH_2_ | a: 2.26, dd (12.6, 4.2) | 1b, 2 | 2, 3, 5, 10, 25 | 11, 25 |
|  |  | b: 1.28 | 1a, 2 | 2, 3, 5, 9, 10, 25 | 3, 11 |
| 2 | 69.2, CH | 3.69, ddd (11.4, 9.6, 4.2) | 1a, 1b, 3 | 1, 3, 4, 10 | 24, 25 |
| 3 | 83.7, CH | 3.00, d (9.6) | 2 | 1, 2, 4, 5, 23, 24 | 1b, 5, 23 |
| 4 | 39.2, C |  |  |  |  |
| 5 | 44.3 CH | 1.38 | 6a, 6b | 1, 3, 4, 6, 7, 9, 10, 23, 24, 25 | 3, 8, 23 |
| 6 | 18.9, CH_2_ | a: 1.73 | 5, 6b, 7a, 7b | 4, 5, 7, 10 |  |
|  |  | b: 1.61 | 5, 6a, 7a, 7b | 4, 5, 7, 10 | 23, 24, 25 |
| 7 | 17.7, CH_2_ | a: 1.64 | 6a, 6b, 7b, 8 | 5, 8, 9 |  |
|  |  | b: 1.35 | 6a, 6b, 7a, 8 | 6, 8 | 25, 26 |
| 8 | 39.8, CH | 2.07 | 7a, 7b, 11 | 7, 9, 11, 13, 14, 26 | 5, 27 |
| 9 | 150.1, C |  |  |  |  |
| 10 | 39.0, C |  |  |  |  |
| 11 | 116.7, CH | 5.34, dt (5.4, 2.4) | 8, 12a, 12b | 9, 10, 12, 13 | 1a, 1b, 25 |
| 12 | 36.7, CH_2_ | a: 1.63 | 11, 12b | 9, 11, 18, 27 | 26 |
|  |  | b: 1.56 | 11, 12a | 9, 11, 13, 14, 27 | 27 |
| 13 | 36.7, C |  |  |  |  |
| 14 | 37.6, C |  |  |  |  |
| 15 | 29.2, CH_2_ | a: 1.42 | 15b, 16a, 16b | 13, 16, 26 |  |
|  |  | b: 1.30 | 15a, 16a, 16b | 13, 14, 16, 17, 26 | 27, 28 |
| 16 | 36.1, CH_2_ | a: 1.69 | 15a, 15b, 16b | 14, 15, 17, 18, 28 | 28, 30 |
|  |  | b: 1.42 | 15a, 15b, 16a | 15, 17, 28 |  |
| 17 | 42.9, C |  |  |  |  |
| 18 | 51.9, CH | 1.56 | 19 | 17, 19, 21, 27, 28 | 21, 26 |
| 19 | 20.1, CH_2_ | 1.35 | 18, 20a, 20b | 17, 18, 20 |  |
| 20 | 28.2, CH_2_ | a: 1.84, tdd (11.4, 9.0, 5.4) | 19, 20b, 21 | 17, 18, 19, 21 | 29 |
|  |  | b: 1.22 | 19, 20a, 21 | 19, 21, 22 |  |
| 21 | 59.6, CH | 0.98, d (9.6) | 20a, 20b, 22 | 17, 20, 22, 28, 29 | 18 |
| 22 | 30.8, CH | 1.46 | 21, 29, 30 | 21, 29, 30 | 28 |
| 23 | 27.9, CH_3_ | 1.00, s |  | 3, 4, 5, 24 | 3, 5, 6b |
| 24 | 16.2, CH_3_ | 0.90, s |  | 3, 4, 5, 23 | 2, 6b, 25 |
| 25 | 26.2, CH_3_ | 1.13, s |  | 1, 5, 9, 10 | 1a, 2, 6b, 7b, 11, 24 |
| 26 | 15.3, CH_3_ | 0.73, s |  | 8, 13, 14, 15 | 7b, 12a, 18 |
| 27 | 15.8, CH_3_ | 0.80. s |  | 12, 13, 14, 18 | 8, 12b, 15b |
| 28 | 14.0, CH_3_ | 0.75, s |  | 16, 17, 18, 21 | 15b, 16a, 22 |
| 29 | 23.0, CH_3_ | 0.83, d (6.6) | 22 | 21, 22, 30 | 20a |
| 30 | 22.1, CH_3_ | 0.89, d (6.6) | 22 | 21, 22, 29 | 16a |

*^a^* The indiscernible signals from overlap or the complex multiplicity are reported without designating multiplicity.

Table S7. NMR assignments for 8 (^1^H for 400 MHz and ^13^C for 100 MHz in pyridine-*d*_5_)

| No. | *δ*_C_, type | *δ*_H_ (*J* in Hz)*^a^* | ^1^H-^1^H COSY | HMBC | ROESY |
| --- | --- | --- | --- | --- | --- |
| 1 | 45.6, CH_2_ | a: 2.39, dd (12.4, 4.4) | 1b, 2 | 2, 3, 5, 10 | 11, 25 |
|  |  | b: 1.59 | 1a, 2 | 2, 3, 10, 25 | 3 |
| 2 | 70.1, CH | 5.59, ddd (12.4, 10.4, 4.0) | 1a, 1b, 3 | 3, 2-COCH_3_ | 24, 25 |
| 3 | 88.6, CH | 3.61, d (10.4) | 2 | 1, 2, 4, 23, 24, 1' | 1b, 5, 23, 1' |
| 4 | 41.5, C |  |  |  |  |
| 5 | 44.3, CH | 1.50 | 6a, 6b | 10, 23, 25 | 3 |
| 6 | 19.2, CH_2_ | a: 1.68 | 5, 6b, 7a, 7b | 8 |  |
|  |  | b: 1.58 | 5, 6a, 7a, 7b | 8, 10 |  |
| 7 | 18.2, CH_2_ | a: 1.63 | 6a, 6b, 7b, 8 | 6, 8 |  |
|  |  | b: 1.34 | 6a, 6b, 7a, 8 |  |  |
| 8 | 40.1, CH | 2.12 | 7a, 7b, 11 | 9, 11, 14, 26 | 27 |
| 9 | 150.8, C |  |  |  |  |
| 10 | 39.0, C |  |  |  |  |
| 11 | 116.8, CH | 5.36 | 8, 12a, 12b | 8, 10, 12, 13 | 1a |
| 12 | 36.9, CH_2_ | a: 1.65 | 11, 12b | 9, 14, 18, 27 |  |
|  |  | b: 1.60 | 11, 12a | 9, 11 |  |
| 13 | 37.0, C |  |  |  |  |
| 14 | 37.9, C |  |  |  |  |
| 15 | 29.4, CH_2_ | a: 1.41 | 15b, 16a, 16b |  |  |
|  |  | b: 1.30 | 15a, 16a, 16b |  |  |
| 16 | 36.5, CH_2_ | a: 1.65 | 15a, 15b, 16b | 14, 17, 18 |  |
|  |  | b: 1.40 | 15a, 15b, 16a | 28 |  |
| 17 | 43.1, C |  |  |  |  |
| 18 | 52.1, CH | 1.52 | 19 | 12, 13, 17, 19, 21, 27, 28 | 26 |
| 19 | 20.3, CH_2_ | 1.33 | 18, 20a, 20b | 18 |  |
| 20 | 28.4, CH_2_ | a: 1.76 | 19, 20b, 21 |  | 28 |
|  |  | b: 1.15 | 19, 20a, 21 |  |  |
| 21 | 59.7, CH | 0.89 | 20a, 20b, 22 | 17 |  |
| 22 | 30.9, CH | 1.40 | 21, 29, 30 |  | 28 |
| 23 | 27.8, CH_3_ | 1.35, s |  | 3, 4, 5, 24 | 3, 1' |
| 24 | 17.4, CH_3_ | 1.20, s |  | 3, 4, 5, 23 | 2 |
| 25 | 26.2, CH_3_ | 1.20, s |  | 1, 5, 9, 10 | 1a, 2 |
| 26 | 15.6, CH_3_ | 0.78, s |  | 8, 13, 14, 15 | 18 |
| 27 | 16.2, CH_3_ | 0.93. s |  | 12, 13, 14, 18 | 8 |
| 28 | 14.2, CH_3_ | 0.78, s |  | 16, 17, 18, 21 | 20a, 22 |
| 29 | 23.2, CH_3_ | 0.87, d (6.4) | 22 | 21, 22, 30 |  |
| 30 | 22.3, CH_3_ | 0.91, d (6.4) | 22 | 21, 22, 29 |  |
| 2-COCH_3_ | 171.0, C |  |  |  |  |
| 2-COCH_3_ | 21.8, CH_3_ | 2.42, s |  | 2-COCH_3_ |  |
| 1' | 106.2, CH | 4.94, d (8.0) | 2' | 3 | 3, 23, 3', 5' |
| 2' | 75.8, CH | 3.98, t (8.4) | 1', 3' | 1', 4' |  |
| 3' | 78.6, CH | 4.23, t (8.8) | 2', 4' | 2', 4' | 1' |
| 4' | 72.1, CH | 4.14, t (8.8) | 3', 5' | 2', 3', 5', 6' |  |
| 5' | 78.1, CH | 3.98 | 4', 6'a, 6'b | 1', 4', 6' | 1' |
| 6' | 63.2, CH_2_ | a: 4.56, dd (11.2, 2.4) | 5', 6'b | 4', 5' |  |
|  |  | b: 4.37, dd (10.8, 5.6) | 5', 6'a | 4', 5' |  |

*^a^* The indiscernible signals from overlap or the complex multiplicity are reported without designating multiplicity.

Table S8. NMR assignments for 9 (^1^H for 600 MHz and ^13^C for 150 MHz in pyridine-*d_5_*)

| No. | *δ*_C_, type | *δ*_H_ (*J* in Hz)*^a^* | ^1^H-^1^H COSY | HMBC | ROESY |
| --- | --- | --- | --- | --- | --- |
| 1 | 45.2, CH_2_ | a: 2.53, dd (12.6, 4.2) | 1b, 2 | 2, 3, 5, 10, 25 | 11, 25 |
|  |  | b: 1.55 | 1a, 2 | 2, 3, 10, 25 | 3 |
| 2 | 73.7, CH | 5.49, td (10.2, 4.2) | 1a, 1b, 3 | 3, 2-COCH_3_ | 24, 25 |
| 3 | 79.6, CH | 3.54, d (10.2) | 2 | 2, 4, 23, 24 | 1b, 5, 23 |
| 4 | 40.4, C |  |  |  |  |
| 5 | 44.7, CH | 1.58 | 6a, 6b | 4, 6, 9, 10, 23, 24, 25 | 3, 8, 23 |
| 6 | 19.4, CH_2_ | a: 1.75 | 5, 6b, 7a, 7b | 5, 7, 10 |  |
|  |  | b: 1.65 | 5, 6a, 7a, 7b | 5 |  |
| 7 | 18.2, CH_2_ | a: 1.67 | 6a, 6b, 7b, 8 | 9, 14 |  |
|  |  | b: 1.35 | 6a, 6b, 7a, 8 | 8 | 26 |
| 8 | 40.1, CH | 2.16, br d (13.8) | 7a, 7b, 11 |  | 5, 27 |
| 9 | 150.9, C |  |  |  |  |
| 10 | 39.4, C |  |  |  |  |
| 11 | 116.8, CH | 5.37 | 8, 12a, 12b | 8, 10, 12, 13 | 1a, 25 |
| 12 | 36.9, CH_2_ | a: 1.67 | 11, 12b | 9, 11, 14, 18 |  |
|  |  | b: 1.58 | 11, 12a | 9, 11, 14 |  |
| 13 | 37.0, C |  |  |  |  |
| 14 | 37.9, C |  |  |  |  |
| 15 | 29.4, CH_2_ | a: 1.42 | 15b, 16a, 16b | 14, 26 |  |
|  |  | b: 1.30 | 15a, 16a, 16b | 13, 14, 26 |  |
| 16 | 36.4, CH_2_ | a: 1.66 | 15a, 15b, 16b | 15, 17, 18, 28 | 30 |
|  |  | b: 1.40 | 15a, 15b, 16a | 14, 17, 18, 21, 28 |  |
| 17 | 43.1, C |  |  |  |  |
| 18 | 52.1, CH | 1.52 | 19 | 12, 13, 17, 19, 21, 27, 28 | 21, 26 |
| 19 | 20.3, CH_2_ | 1.30 | 18, 20a, 20b | 17, 18, 20 |  |
| 20 | 28.4, CH_2_ | a: 1.75 | 19, 20b, 21 | 17, 19, 21 | 30 |
|  |  | b: 1.16 | 19, 20a, 21 |  |  |
| 21 | 59.6, CH | 0.90 | 20a, 20b, 22 | 17, 18, 22, 28, 29, 30 | 18 |
| 22 | 30.9, CH | 1.39 | 21, 29, 30 | 21, 29, 30 |  |
| 23 | 28.5, CH_3_ | 1.25, s |  | 3, 4, 5, 24 | 3, 5 |
| 24 | 16.9, CH_3_ | 1.21, s |  | 3, 4, 5, 23 | 2 |
| 25 | 26.2, CH_3_ | 1.28, s |  | 1, 5, 9, 10 | 1a, 2, 11 |
| 26 | 15.6, CH_3_ | 0.80, s |  | 8, 13, 14, 15 | 7b, 18 |
| 27 | 16.1, CH_3_ | 0.90. s |  | 12, 13, 14, 18 | 8 |
| 28 | 14.2, CH_3_ | 0.76, s |  | 16, 17, 18, 21 |  |
| 29 | 23.2, CH_3_ | 0.86, d (6.6) | 22 | 21, 22, 30 |  |
| 30 | 22.3, CH_3_ | 0.91, d (6.6) | 22 | 21, 22, 29 | 16a, 20a |
| 2-COCH_3_ | 170.8, C |  |  |  |  |
| 2-COCH_3_ | 21.4, CH_3_ | 2.03, s |  | 2-COCH_3_ |  |

*^a^* The indiscernible signals from overlap or the complex multiplicity are reported without designating multiplicity.

Supplementary Figures

Figure S1. Representative enfumafungin-type antibiotics.

Figure S2. Construction of the *∆ku80* mutant *A. oryzae* J001.

(A) The *ku80*-cutting Cas9 plasmid and the *ku80*-disrupting donor plasmid. (B) Disruption of the *ku80* gene in *A. oryzae* S184 and validation of the *∆ku80* mutant transformant via PCR analysis.

Figure S3. Comparison of the site-specific integration efficiency of *A. oryzae* S184 and *A. oryzae* J001.

Figure S4. Scheme of the counter-selection process to eliminate the *pyrG* selection marker.

(A) Conventional method using the non-selective plate medium; (B) Refined strategy using the selective liquid.

Figure S5. Amino acid sequence identities of the proteins encoded by the gene clusters *fso*, *efu*, and *efm*.

Figure S6. Functional analysis of the fusion enzyme FsoA_(TC)_EfuA_(GT)_.

HPLC-MS analysis was carried out using a Vanquish HPLC system (Thermo Scientific, USA) and an TSQ Quantis Plus ion trap mass spectrometer coupled with an atmospheric pressure chemical ionization (APCI) source (Thermo Scientific, USA).

Figure S7. Amino acid sequence alignment between EfuH2 and FsoD.

Figure S8. Amino acid sequence alignment between EfuH1 and FsoF.

Figure S9. Amino acid sequence alignment between EfuG and FsoE.

Figure S10. In vitro enzymatic assay of EfuI.

(A) SDS-PAGE analysis of the purified MBP-tagged EfuI; (B) HPLC profiles of the reaction mixtures obtained by incubating 6 with EfuI or inactive EfuI; (C) HPLC profiles of the reaction mixtures obtained by incubating fuscoatroside with EfuI or inactive EfuI; (D) Extracted ion chromatograms of the reaction mixtures obtained by incubating fuscoatroside with EfuI or inactive EfuI.

Figure S11. Reconstitution of enfumafungin biosynthesis in *A. oryzae* through combination of *efuA_(TC)_fsoA_(GT)_*, *fsoD*, *fsoE*, *fsoF*, and *efuI*.

Figure S12. Elucidation of the catalytic sequence of FsoA_(GT)_ in the biosynthesis of fuscotraside via heterologous expression in *A. oryzae.*

HPLC-MS analysis was carried out using a Vanquish HPLC system (Thermo Scientific, USA) and an TSQ Quantis Plus ion trap mass spectrometer coupled with an atmospheric pressure chemical ionization (APCI) source (Thermo Scientific, USA).

Figure S13. NMR spectra of 5.

(A) ^1^H NMR spectrum in pyridine-*d*_5_ at 600 MHz; (B) ^13^C NMR spectrum in pyridine-*d*_5_ at 150 MHz; (C) ^1^H-^1^H COSY spectrum in pyridine-*d*_5_ at 600 MHz; (D) HSQC spectrum in pyridine-*d*_5_ at 600 MHz; (E) HMBC spectrum in pyridine-*d*_5_ at 600 MHz; (F) ROESY spectrum in pyridine-*d*_5_ at 600 MHz.

Figure S14. NMR spectra of 6.

(A) ^1^H NMR spectrum in pyridine-*d*_5_ at 600 MHz; (B) ^13^C NMR spectrum in pyridine-*d*_5_ at 150 MHz; (C) ^1^H-^1^H COSY spectrum in pyridine-*d*_5_ at 600 MHz; (D) HSQC spectrum in pyridine-*d*_5_ at 600 MHz; (E) HMBC spectrum in pyridine-*d*_5_ at 600 MHz; (F) ROESY spectrum in pyridine-*d*_5_ at 600 MHz.

Figure S15. NMR spectra of 7.

(A) ^1^H NMR spectrum in CDCl_3_ at 600 MHz; (B) ^13^C NMR spectrum in CDCl_3_ at 150 MHz; (C) ^1^H-^1^H COSY spectrum in CDCl_3_ at 600 MHz; (D) HSQC spectrum in CDCl_3_ at 600 MHz; (E) HMBC spectrum in CDCl_3_ at 600 MHz; (F) ROESY spectrum in CDCl_3_ at 600 MHz.

Figure S16. NMR spectra of 8.

(A) ^1^H NMR spectrum in pyridine-*d*_5_ at 400 MHz; (B) ^13^C NMR spectrum in pyridine-*d*_5_ at 100 MHz; (C) ^1^H-^1^H COSY spectrum in pyridine-*d*_5_ at 400 MHz; (D) HSQC spectrum in pyridine-*d*_5_ at 400 MHz; (E) HMBC spectrum in pyridine-*d*_5_ at 400 MHz; (F) ROESY spectrum in pyridine-*d*_5_ at 400 MHz.

Figure S17. NMR spectra of 9.

(A) ^1^H NMR spectrum in pyridine-*d*_5_ at 600 MHz; (B) ^13^C NMR spectrum in pyridine-*d*_5_ at 150 MHz; (C) ^1^H-^1^H COSY spectrum in pyridine-*d*_5_ at 600 MHz; (D) HSQC spectrum in pyridine-*d*_5_ at 600 MHz; (E) HMBC spectrum in pyridine-*d*_5_ at 600 MHz; (F) ROESY spectrum in pyridine-*d*_5_ at 600 MHz.

Reference
